# Lessons from the similarities and differences in fluid resuscitation between burns and sepsis: a bibliometric analysis

**DOI:** 10.3389/fmed.2025.1561619

**Published:** 2025-03-04

**Authors:** Dongxu Zhou, LuLu He, Wei Shi, Penglin Ma

**Affiliations:** Department of Critical Care Medicine, Guiqian International General Hospital, Guiyang, China

**Keywords:** burn, sepsis, resuscitation, outcomes, complications, bibliometric analysis

## Abstract

**Background:**

Fluid is an essential component of initial resuscitation in sepsis or burns. Meanwhile, the optimal strategy of titrating fluids for both of the two conditions remains uncertain. In this bibliometric analysis, we compared the similarities and differences in fluid resuscitation between sepsis and burns in recent publications.

**Methods:**

Literatures related to fluid resuscitation in either sepsis or burns were searched in the Web of Science database Core Collection from January 1, 1992, to December 31, 2022. CiteSpace and VOSviewer was used for bibliometric analysis.

**Results:**

A total of 1,549 and 468 publications on fluid resuscitation in sepsis and burns were retrieved from 1992 to 2022. Based on the occurrences, 341 and 86 high-frequency keywords were screened out from sepsis and burns publications, respectively, which were similarly categorized into 5 clusters [i.e. “mechanisms of hypovolemia” (cluster 1), “titration of fluid” (cluster 2), “outcomes or complications” (cluster 3), “pathophysiological alternations” (cluster 4), and “fluid types and others” (cluster 5)]. The high-frequency keywords of the top 20 were more concentrated in cluster 3 and cluster 2, with “mortality” ranked the top in both sepsis and burns literature. Significantly, 3 keywords in cluster 2 ranked in the top 5, including “goal directed resuscitation” (the 3rd), fluid responsiveness (the 4th) and fluid balance (the 5th) in sepsis literature, while the keywords of “microvascular exchange” (cluster 1) and “abdominal compartment syndrome” (ACS, cluster 3) ranked at the second and the fifth place in burns publications. Keyword burst analysis demonstrated that the keyword with the highest burst strength (BS) was “formula” (BS = 5.88, 2008–2014), followed by management (BS = 4.79, 2012–2022), ACS (BS = 4.76, 2006–2010), and fluid creep (BS = 4.74, 2011–2016) in burn publications, but they were dobutamine (BS = 12.31, 1992–2008), cardiac output (BS = 9.79, 1993–2001), catecholamine (BS = 9.54, 1993–2006), and consumption (BS = 7.52, 1992–2006) in sepsis literature. Moreover, the most frequently cited article in either sepsis or burns was categorized into cluster 2, that investigated goal-directed fluid therapy for sepsis and formula improvement for burns resuscitation.

**Conclusion:**

It was demonstrated that the research priorities in titrating fluid were mainly concentrated on targeting hemodynamics in sepsis vs. improving formula (which briefly calculates the increased microvascular permeability) in burns, while concerning of “outcomes and complications” in fluid resuscitation similarly after 1992. However, hemodynamics and microvascular permeability have been simultaneously well considered in few previous studies regarding fluid resuscitation in either sepsis or burns.

## Introduction

Administering adequate fluid to improve circulation, perfusion, and oxygen delivery is a common goal of physicians during initial resuscitation in patients with sepsis or burns, both of which share similar pathophysiological mechanisms of hypovolemia (1, 2). However, a growing body of evidence suggests that the potential harms of fluid accumulation are well documented (3, 4). Of note, severe complications of overload such as hypoxia, abdominal compartment syndrome (ACS) and tissue edema could counterbalance the benefits of fluid resuscitation. Therefore, optimizing fluid therapy has been therefore one of the research priorities in sepsis and burns in the past three decades.

Severe burns are usually administered with a large amount of fluids within the first 24 h because of burn shock, characterized by specific microvascular and hemodynamic changes (5). In the past few decades, efforts have been made to promote formulas for estimating fluid requirement based on total burn area (6). In addition, goal-directed fluid therapy has been comprehensively investigated in the initial fluid resuscitation of severe burns since the report of Dries and Waxman in 1991 (7). Meanwhile, there is no consensus on the optimization of initial fluid resuscitation in severe burns. It is worth noting that “fluid creep,” a phenomenon of excessive fluid demand, resulting in a series of harmful effects (8), has attracted increasing attention in acute burn resuscitation recently (9–11).

Initial fluid resuscitation is an essential component of intensive therapies for sepsis (12). The therapeutic goal is to rapidly reverse hypoperfusion (with or without hypotension), administering fluid boluses, continuing fluid replacement and frequently using vasopressors as well (13). This approach was encouraged worldwide due to the results of Rivers's EGDT trial (Early Goal-Directed Therapy) (14) and high recommendation of the “Surviving Sepsis Campaign Guidelines for Management of Severe Sepsis and Septic Shock” (15). However, the recommended goal of fluid resuscitation did not always represent the optimal volume of an individual need, because of interindividual variability resulted from by various biometric factors (16). Accordingly, it was found that overload was common in clinical practices although the cardiac output could be improved large probably by an aggressive fluid resuscitation strategy in the early phase of sepsis (2). Moreover, positive fluid balance during initial resuscitation was associated with higher morbidity and mortality of sepsis (17). Therefore, the latest update of the Surviving Sepsis Campaign (SSC) international guidelines proposed the physiology-based individualized initial fluid resuscitation (18).

In fact, it remains problematic to avoid overload while ensuring adequate plasma volume expansion during fluid resuscitation in both sepsis and burns. Aimed at finding useful information to suggest further research priorities, this bibliometric analysis described the similarities and differences in recent publications on fluid resuscitation between sepsis and burns.

## Materials and methods

### Data sources and search strategies

**Figure 1:**
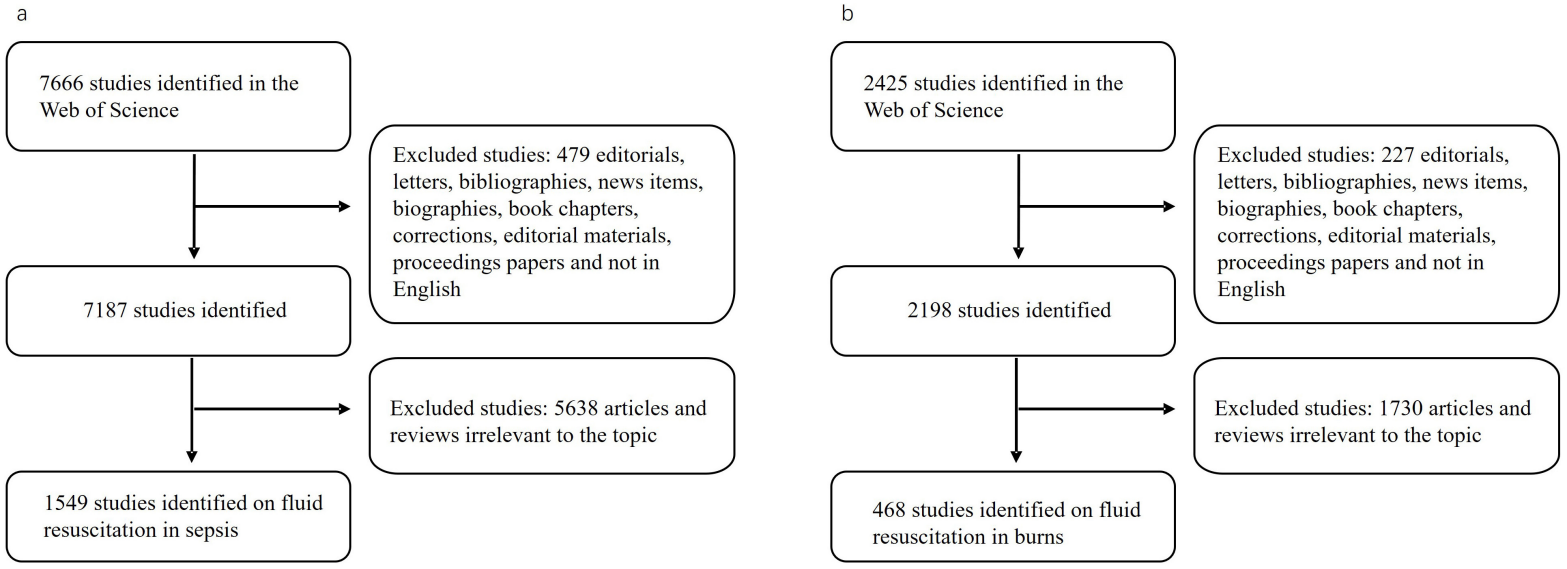
Flowchart for publication selection. The search strategy and process of selecting publications on fluid resuscitation for sepsis **(A)** and burn **(B)**.

Literature search was performed in the Web of Science (WoS) database Core Collection, which is one of the most comprehensive, systematic and authoritative databases, and has been successfully used for bibliometric analysis (19). The type of literature was limited to original articles and reviews. Publications on fluid resuscitation in burns or sepsis from 1992 to 2022 were retrieved for this bibliometric analysis. The retrieval strategies were as follows: (TS = (burn OR thermal injury) AND TS = (fluid OR liquid OR volume OR colloid OR crystalloid) AND TS = (resuscitation OR therapy OR replacement OR balance OR responsiveness OR challenge)) for burn, and (TS = (sepsis OR septic OR bacteremia OR septicemia OR endotoxemia) AND TS = (fluid OR liquid OR volume OR colloid OR crystalloid) AND TS = (resuscitation OR therapy OR replacement OR balance OR responsiveness OR challenge)) for sepsis, respectively. To avoid changes in publication and citation due to frequent database updates, all searches were performed on a single day, i.e., July 31th, 2023. In addition, all data were obtained from this public database and did not involve any personal information. Therefore, the informed consent for this study was waived by the Ethics Committee of Guiqian International General Hospital. The detailed procedure of literature selection and screening was shown in Figures 1A, B.

### Data collection

Two researchers (Dongxu Zhou and Lulu He) independently searched the literature. The consistency of the results was 96.8%, which is significant. The title, abstract, authors, countries, institutions, journal, publication year, key words and the total/average citation of the literature were screened and recorded. Reassessment was performed by Dr. Penglin Ma and a three-way coordination was performed immediately if there was any discrepancy in publications screening between the two researchers. The flow chart of publication selection is shown in Figure 1. VOSviewer version 1.6.16, and CiteSpace version 5.8.R1 64 bit, was used to present, analyze, and describe the data.

### Bibliometric analysis

Keywords represent concise summaries of the core content of the literature. They can reveal the overall characteristics of research results, the inherent connections between research topics, the development trajectory of academic research, and its directions. Therefore, high-frequency keywords can reflect the research hotspots and main research content in the field. In this study, the scientific mapping of keyword co-occurrences and co-cited references in the recruited publications were completed using VOSviewer 1.6.16, a free Java-based software that is mainly oriented to bibliographic data and focuses on the visualization of scientific knowledge and was developed by Nees Jan van Eck and Ludo Waltman in 2009 (20). In the network map, different nodes represent different keywords or citation of publications, and the node size reflects the frequency of occurrence of the keyword or the number of citations of the publication (21). Links between nodes indicate relationships such as co-reference, co-occurrence, and collaboration. Furthermore, VOSviewer can classify keywords into different clusters based on co-occurrence analysis, and simultaneously color them according to the time course. The definition of average appearing year (AAY) was used to quantify the relative novelty of a keyword (22). CiteSpace 5.8.R1, another software developed by professor Chaomei Chen, was used to identify new trends and advances in the scientific literature (23). In our study, it was mainly used for keyword bursts analysis.

## Results

### Trends in global publication growth

**Figure 2:**
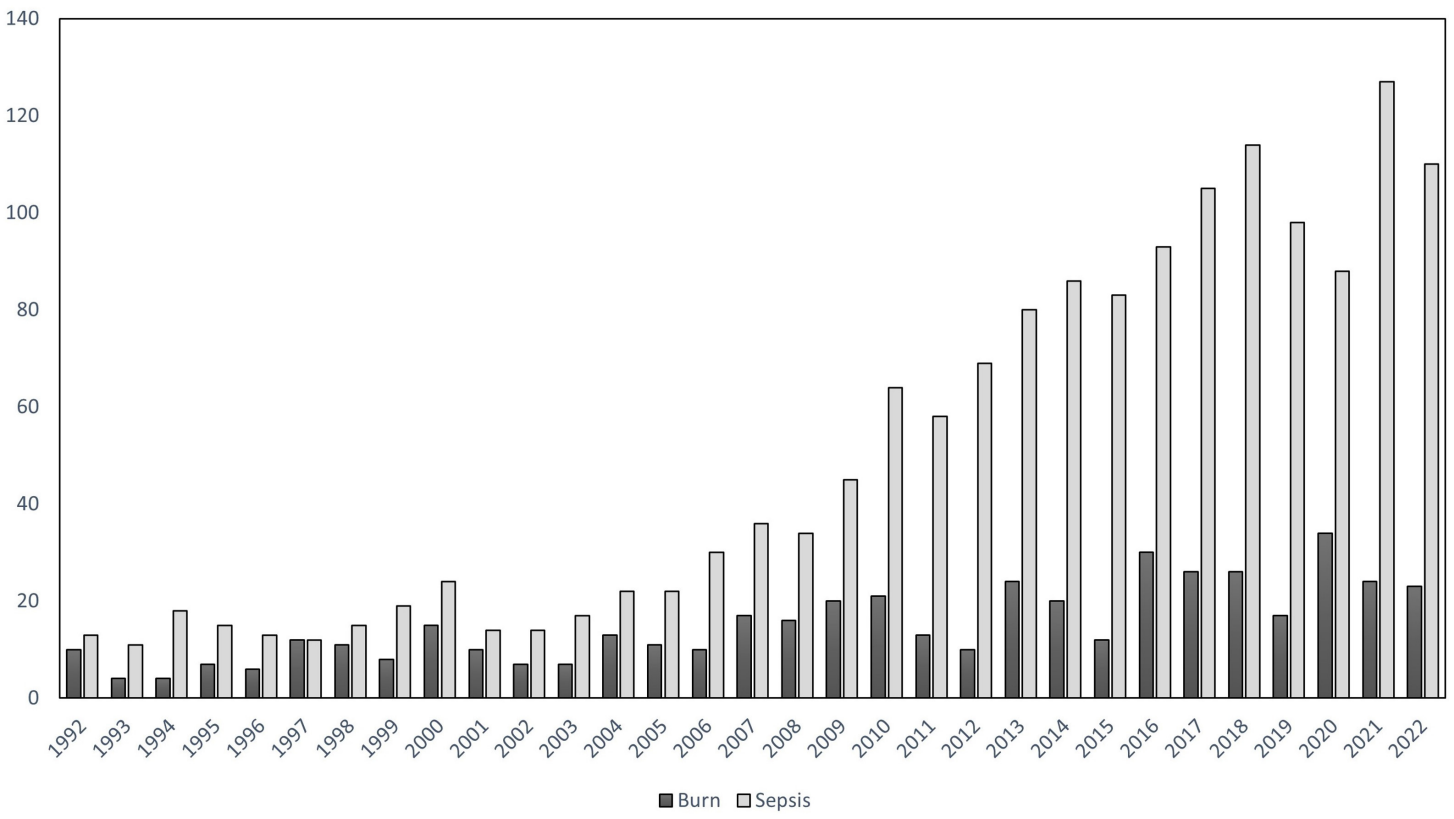
Annual publications from 1992 to 2022. The X-axis represented each year from 1992 to 2022, the Y-axis represented the number of annual publications on fluid resuscitation for burn (dark bar) and sepsis (gray bar).

There have been 1,549 publications on sepsis fluid resuscitation since 1992 (Figure 2). The number of publications per year rapidly increased overtime, from about 20 articles in 1992, to more than 50 in 2010, and above 100 after 2021. A total number of 468 publications on fluid resuscitation in burn researches met the inclusion criteria for this bibliometric analysis. The annual number of publications never exceeded 50 until 2022 in this field. Meanwhile, it was found that the cumulative proportions were significantly increased in publications on fluid resuscitation among all literatures in both sepsis and burns over years (Supplementary Figure S1).

### Keywords mapping analysis

VOSviewer was used to cluster the keywords of the included literature. Subject terms, synonyms, repetitive words and unrelated terms (sepsis, septic shock, severe sepsis, burn, burns, injury, thermal injury, burn injury, trauma, resuscitation, therapy, fluid therapy, fluid resuscitation, advance, association, critical illness, etc.) were excluded. A mapping analysis was performed for the keywords that occurred equal to or more than 5 times either in sepsis or burns of the literature.

**Figure 3:**
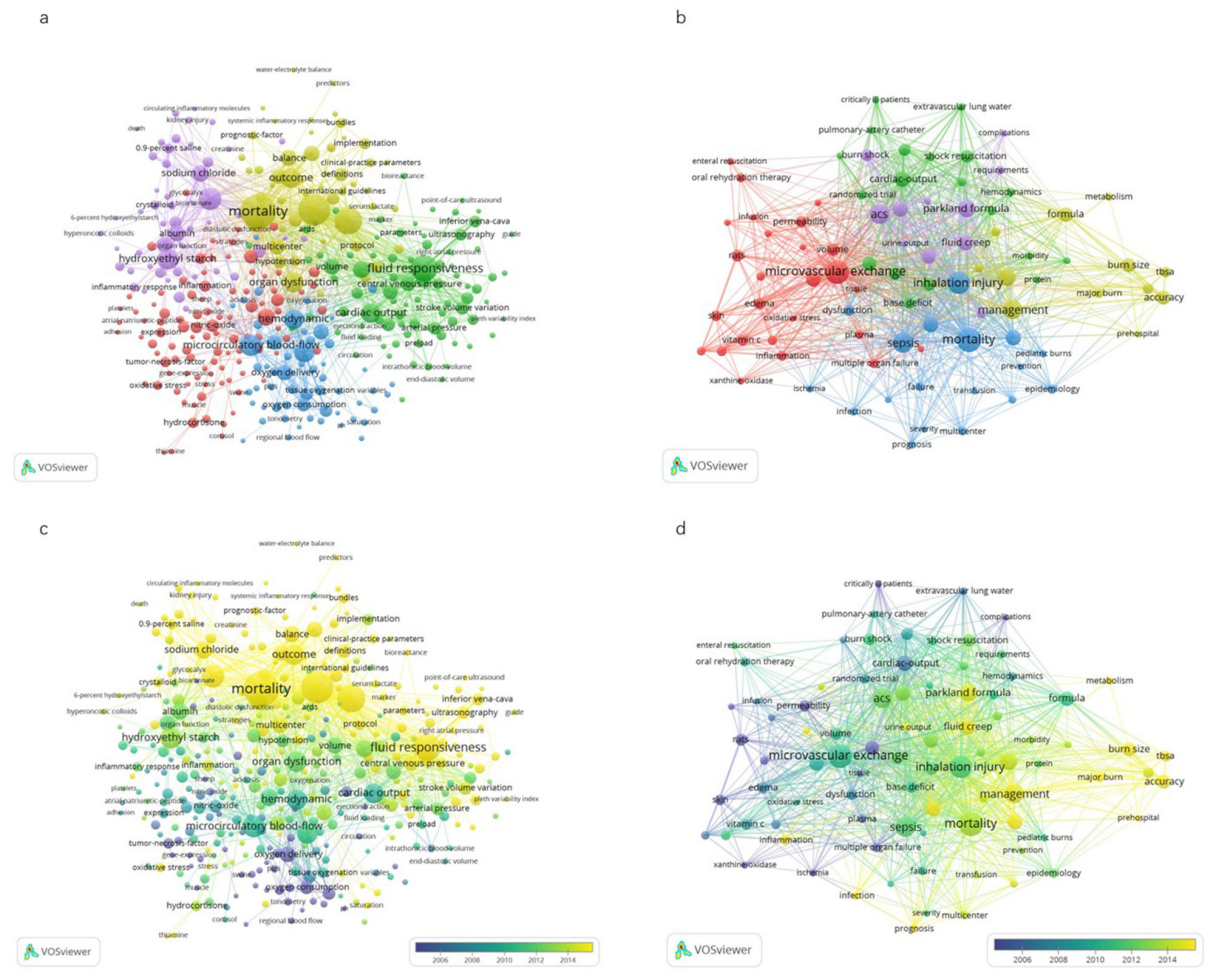
Visualization map of Keywords co-occurrence network in publications. The figure represented the network of keywords with more than 5 occurrences in publications on fluid resuscitation for sepsis **(A)** and burn **(B)**. The size of the circles indicated the co-occurrence frequency of keywords. The clusters of the keywords (i.e., a set of keywords calculated in the co-occurrence network as a community) were showed in different colors. The connecting lines indicated co-occurrence of the 2 keywords at both ends. The thickness of lines between circles indicated strength of linkage calculated by the frequency of co-occurrence. In addition, VOSviewer colored all keywords in accordance with the average time when the keyword appeared in publications for sepsis **(C)** and for burn **(D)**. The blue color represented the keywords appeared relatively earlier upon time course, while keywords in yellow for recent appearance.

As shown in Figure 3A, 341 keywords from 1,549 publications on fluid resuscitation in sepsis were analyzed (listed in Supplementary Figure S1), resulting in 5 different clusters. Cluster 1 (100 Keywords) was closely related to the mechanisms of sepsis-induced hypovolemia (red), including high-frequency keywords such as vascular dysfunction (76 occurrences), nitric oxide (42 occurrences) and inflammation (33 occurrences). Eighty-two keywords in cluster 2 were mainly related to titration of fluid therapy, as indicated in green, including fluid responsiveness (225 occurrences), cardiac output (107 occurrences) and blood pressure (77 occurrences), etc. In cluster 3, 52 keywords were related to outcomes or complications shown in yellow, that was weighted the highest in occurrences, such as mortality (426 occurrences), management (331 occurrences), and acute kidney injury (AKI; 148 occurrences). Cluster 4 (56 keywords) was related to pathophysiological changes in fluid resuscitation shown in blue, including microcirculatory blood-flow (140 occurrences), norepinephrine therapy (122 occurrences) and hemodynamic (110 occurrences), etc. The remaining 51 out of 341 keywords were categorized into the cluster of fluid types and others (cluster 5), including hydroxyethyl starch (128 occurrences), sodium chloride (77 occurrences) and albumin (70 occurrences, purple circles), etc.

As show in Figure 3B, a total of 86 keywords extracted in 468 publications on burn fluid resuscitation was also categorized in 5 distinct clusters, which were similar to that in sepsis. Keywords in cluster 1 were largely related to the mechanism of burn shock (26 keywords in red). Cluster 2 was the keywords associated with titration of fluid therapy (19 keywords in green). Keywords in cluster 3 were related to complications and outcomes (19 keywords in blue, with the highest weight in occurrences). Cluster 4 was comprised of the keywords with regard to pathophysiological alternation in fluid resuscitation (12 keywords in yellow). The last cluster was related to the keywords for fluid type and the others (10 keywords in purple).

In addition, as shown in Figures 3C, D, VOSviewer colors all keywords according to their average time of appearance. Blue indicates keywords appearing relatively early in the time course (i.e., earliest, before 2006), and yellow indicates keywords that appear more recently (i.e., latest, after 2014). In the literature of fluid resuscitation in burns, the top 3 high-frequency keywords appeared earliest vs. latest were cardiac-output, oxygen delivery, permeability vs. management, formula and critical care. Meanwhile, the earliest vs. the latest top 3 high-frequency keywords were oxygen delivery, oxygen consumption and dobutamine vs. mortality, management, goal directed resuscitation in the literature in sepsis (Supplementary Table S2).

### Analyses of the top 20 high-frequency keywords

The top 20 high-frequency keywords in the literature of fluid resuscitation for burns and sepsis were similarly distributed into 5 clusters, as shown in Table 1. Of the top 20, 6 and 5 keywords were clustered into “outcomes or complications” in sepsis literature [mortality (the 1st), AKI (the 6th), outcome (the 9th), organ dysfunction (the 11th) and survival (the 14th)], and in burns literature including mortality (the 1st), sepsis (the 4th), ACS (the 5th), critical care (the 7th), AKI (acute kidney injury, the 10th) and outcomes (the 13th), respectively. Of the top 20, there were 6 keywords in sepsis [goal directed resuscitation (the 3rd), fluid responsiveness (the 4th), fluid balance (the 5th), cardiac output (the 13th), blood pressure (the 15th) and volume (the 20th)], and 4 keywords in burns [Parkland formula (the 8th), cardiac-output (the 9th), formula (the 14th) and blood pressure (the 15th)] in the cluster of “titration of fluid therapy.” There were 1 and 3 out of top 20 keywords clustered into “mechanisms of hypovolemia” and “pathophysiological alternations,” respectively in literature of both sepsis and burns. The other 6 and 5 of top 20 high frequency keywords were classified into “fluid types and others” in burns and sepsis literature. Notably, mortality was the top ranked keyword in either burns or sepsis literature. In addition, it was demonstrated that three keywords in the cluster of “titration of fluid therapy” including goal directed resuscitation (the 3rd), fluid responsiveness (the 4th) and fluid balance (the 5th) were ranked in top five in sepsis literature. Meanwhile, the keywords of “microvascular exchange” and “abdominal compartment syndrome” (ACS) ranked at the second and the fifth place in burns publications.

**Table 1:** Classification of the top 20 high-frequency keywords in publications on fluid resuscitation for sepsis and burns.

**Rank**	**Sepsis**				**Rank**	**Burn**			
	**AAY**	**Keywords**	**Occurrences**	**Total link strength**		**AAY**	**Keywords**	**Occurrences**	**Total link strength**
Cluster 1. Mechanisms of hypovolemia					Cluster 1. Mechanisms of burn shock				
17th	2011.2	Vascular dysfunction	76	361	2nd	2010.1	Microvascular exchange	61	178
Cluster 2. Titration of fluid					Cluster 2. Titration of fluid				
3rd	2016.0	Goal directed resuscitation	239	1,112	8th	2014.5	Parkland formula	32	97
4th	2016.0	Fluid responsiveness	225	1,155	9th	2006.4	Cardiac-output	26	82
5th	2017.6	Fluid balance	120	534	14th	2011.0	Formula	23	70
13th	2009.5	Cardiac output	107	540	15th	2009.4	Blood pressure	22	54
15th	2012.6	Blood pressure	77	389					
20th	2011.9	Volume	69	357					
Cluster 3. Outcomes or complications					Cluster 3. Outcomes or complications				
1st	2016.3	Mortality	426	1,772	1^st^	2013.3	Mortality	63	206
6th	2017.2	AKI	148	764	4th	2011.6	Sepsis	46	164
9th	2017.3	Outcome	124	513	5th	2012.3	ACS	41	132
11th	2012.0	Organ dysfunction	118	542	7th	2014.0	Critical care	33	98
14th	2014.3	Survival	87	418	10th	2015.8	AKI	26	82
					13th	2015.9	Outcomes	24	81
Cluster 4. Pathophysiological alternations					Cluster 4. Pathophysiological alternations				
7th	2009.9	Microcirculatory blood-flow	140	713	11th	2015.5	Fluid creep	25	82
10th	2010.9	Norepinephrine therapy	122	685	18th	2005.1	Oxygen delivery	20	73
12th	2009.5	Hemodynamic	110	629	20th	2011.7	Serum lactate	17	69
Cluster 5. Fluid types and others					Cluster 5. Fluid types and others				
2nd	2016.5	Management	331	1,507	3rd	2011.4	Inhalation injury	54	168
8th	2013.0	Hydroxyethyl starch	128	651	6th	2014.6	Management	40	118
16th	2015.8	Sodium chloride	77	370	12th	2012.8	Colloid resuscitation	25	102
18th	2012.2	Albumin	70	361	16th	2017.0	Accuracy	21	47
19th	2014.6	Multicenter	70	327	17th	2017.4	Burn size	21	42
					19th	2012.8	Albumin	20	58

Occurrences were calculated on occurring frequency of the keywords in the literature. Total link strength: Links between nodes can be cooperative relationships, co-occurrence relationships, reference relationships, etc., and each link has a weight or strength value. Total link strength is the sum of the strength of all the links connecting two nodes. AAY, Average appearing years [the average publication year of the articles in which the keyword occurs (to the nearest 1 decimal place)]. AKI, acute kidney injury; ACS, abdominal compartment syndrome.

### Keyword burst analysis in literature of fluid resuscitation for burn and sepsis

**Figure 4:**
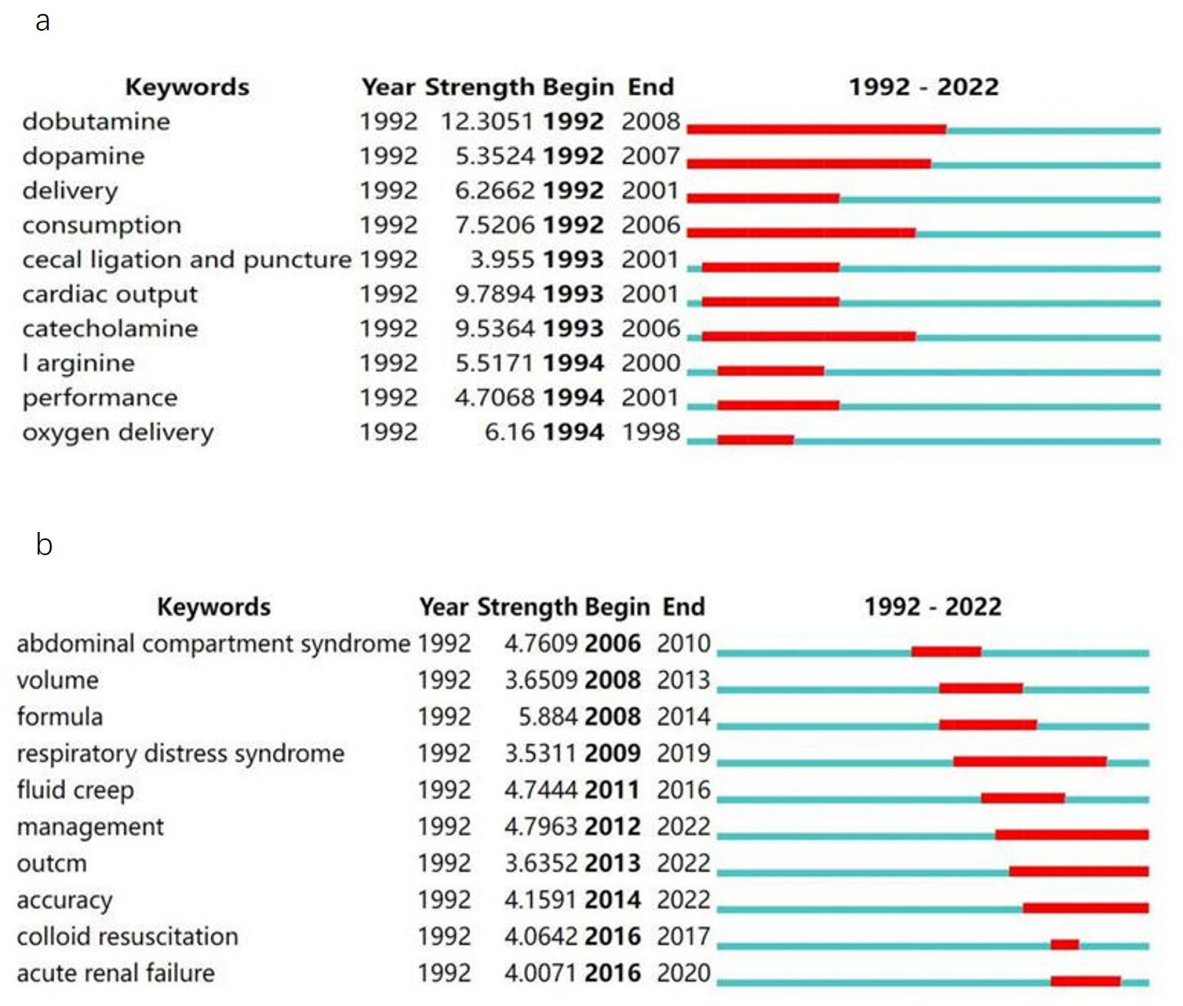
Top 10 keywords with the strongest citation bursts. This represented the keywords with the strongest citation bursts in publications on fluid resuscitation for sepsis **(A)** and for burn **(B)** from 1992 to 2022. The time interval of a burst was marked as a red section on the blue timeline to indicate the beginning/ending year and the duration of a citation burst.

Burst detection identified the emerging concepts that caught the attention of peer investigators. Keywords bursts indicated possible hot research topics. The timeline was represented by a blue line divided by year, and the red part of the blue timeline represents the time interval of the burst, indicating the start/end year and duration of a citation burst. Figures 4A, B show the keyword burst analysis in publications related to fluid resuscitation in sepsis and burn.

Keywords burst analysis was performed in 1,549 articles on sepsis fluid resuscitation in the WoS database from 1992 to 2022. Dobutamine had the highest burst strength (burst strength: 12.3051), and lasted from 1992 to 2008, followed by cardiac output (burst strength: 9.7894, duration: 1993–2001), catecholamine (burst strength: 9.5364, duration: 1993–2006), and consumption (burst strength: 7.5206, duration: 1992–2006).

A total of 468 articles on burn fluid resuscitation from 1992 to 2022 were searched in the WOS database, and an outbreak of keywords was detected. The keyword with the highest burst strength was “formula” (burst strength: 5.884), which occurred from 2008 till 2014, followed by management (burst strength: 4.7963, duration: 2012–2022), abdominal compartment syndrome (burst strength: 4.7609, duration: 2006–2010), and fluid creep (burst strength: 4.7444, duration: 2011–2016).

### Highly cited literature analysis

Highly cited articles are the foundation of a research field and reflect important scientific achievements and academic influence to a certain extent. The co-citation graph of cited references is displayed at Supplementary Figure S2. Table 2 list the top 10 highly cited articles on fluid resuscitation for burn and sepsis, respectively.

**Table 2:** Top 10 highly cited publications on burns and sepsis fluid resuscitation.

**Ranking**	**Total citations**	**Year**	**Titles**	**Author**	**Publication type**
**Top 10 articles on burns fluid resuscitation**					
1	89	1968	Physiological response to crystalloid resuscitation of severe burns.	Baxter CR	Prospective study
2	81	2000	Protection from excessive resuscitation: “pushing the pendulum back”	Pruitt BA Jr	Reviews
3	67	2007	The phenomenon of “fluid creep” in acute burn resuscitation	Saffle JI	Reviews
4	61	2007	The association between fluid administration and outcome following major burn: a multicenter study	Klein MB	Retrospective cohorts study
5	60	2002	How well does the Parkland formula estimate actual fluid resuscitation volumes?	Cartotto RC	Retrospective cohorts study
6	59	2000	Intra-abdominal hypertension and abdominal compartment syndrome in burn patients	Ivy ME	Prospective study
7	54	2008	American Burn Association practice guidelines burn shock resuscitation.	Pham TN	Guidelines
8	53	2000	A biopsy of the use of the Baxter formula to resuscitate burns or do we do it like Charlie did it?	Engrav LH	Retrospective Cohorts study
9	50	1974	Fluid volume and electrolyte changes of the early postburn period	Baxter CR	Reviews
10	41	2004	A clinical randomized study on the effects of invasive monitoring on burn shock resuscitation	Holm C	RCT
**Top 10 Articles on sepsis fluid resuscitation**					
1	528	2001	Early goal-directed therapy in the treatment of severe sepsis and septic shock	Rivers E	RCT
2	229	2013	Surviving sepsis campaign: international guidelines for management of severe sepsis and septic shock	Dellinger RP	Guidelines
3	223	2011	Fluid resuscitation in septic shock: a positive fluid balance and elevated central venous pressure are associated with increased mortality	Boyd JH	RCT
4	217	2016	The third international consensus definitions for sepsis and septic shock (sepsis-3)	Singer M	Reviews
5	190	2006	Comparison of two fluid-management strategies in acute lung injury	Wiedemann HP	RCT
6	184	2014	Goal-directed resuscitation for patients with early septic shock	Peake SL	RCT
7	181	2017	Surviving sepsis campaign: international guidelines for management of sepsis and septic shock: 2016.	Rhodes A	Guidelines
8	152	2001	Epidemiology of severe sepsis in the United States: analysis of incidence, outcome, and associated costs of care	Angus DC	Retrospective Cohorts study
9	152	2014	A randomized trial of protocol-based care for early septic shock.	Yealy DM	RCT
10	147	1992	Definitions for sepsis and organ failure and guidelines for the use of innovative therapies in sepsis.	Bone RC	Reviews

RCT, Randomized controlled trial.

There were only 1 randomized controlled trial (RCT) (24) and 2 prospective studies (10, 25), in addition to 3 reviews (8, 26, 27), 3 retrospective cohort studies and 1 clinical practice guideline among the top 10 highly cited articles on burn fluid resuscitation (9, 28–30). The most frequently cited article (with 89 citations) was “Physiological response to crystalloid resuscitation of severe burns” (23). The next was “Protection from Excessive Resuscitation: “Pushing the Pendulum Back”” (26) with 81 citations. It was noted that the latest highly cited article was the updated American Burn Association Practice Guidelines for Burn Shock Resuscitation, which was published in 2008 (30).

Table 2 lists top 10 highly cited articles on sepsis fluid resuscitation researches from 1992 to 2022, including 5 randomized controlled trials, 2 clinical guidelines, 2 reviews (updating sepsis definition), and 1 retrospective cohort studies. The most frequently cited article (with 528 citations) was “Early goal-directed therapy in the treatment of severe sepsis and septic shock” (14). This was followed by “Surviving Sepsis Campaign: International Guidelines for Management of Severe Sepsis and Septic Shock” (31), with 229 citations. The 10 highly citated articles were published from 1992 to 2017.

### Fluid resuscitation recommendations for burn and sepsis in updated guidelines

A total of 4 international practice guidelines for burn care were retrieved in this study (Table 3) (30, 32–34). Two of them were conducted by American Burn Association (30, 32). Of note, the formula of starting acute fluid resuscitation with 2 mL/kg/% TBSA (total body surface area) burn plus considering use of albumin is recommended to reduce the total volume of resuscitation in the recently updated American Burn Association Clinical Practice Guidelines on Burn Shock Resuscitation (32).

**Table 3:** Recommendations for fluid resuscitation in burns and sepsis guidelines.

**Year**	**Authors**	**Title**	**Resuscitation plan**
**Burn guidelines**			
2008	Tam N. Pham	American burn association practice guidelines burn shock resuscitation	**Recommendation:** 1. Current recommendations are to initiate formal fluid resuscitation when burns>20% TBSA, preferably through the intravenous route. 2. There are no available level I or level II publications to guide the choice of isotonic crystalloid resuscitation. 3. The Baxter formula is the most commonly used formula at U.S. 4. Hypertonic saline resuscitation should be reserved for experienced burn physicians. 5. Resuscitation formulas are useful as starting guidelines, rather than rigid goals for volume resuscitation. **Objective:** 1. Titration of fluids to maintain renal perfusion to obtain a urinary output of 0.5 ml/kg/h is considered adequate for adults, whereas a urinary output of 1 ml/kg/h is an appropriate target for young pediatric patients.
2016	Yuichiro	The wound/burn guidelines−6: Guidelines for the management of burns	**Recommendation:** 1. Remarks on recommendation: It is recommended to initiate fluid resuscitation using the Parkland method. **Objective:** The urine volume is recommended as an index for the infusion rate. The infusion rate should be adjusted to maintain the urine volume at 0.5 mL/kg per h or 30–50 mL/h or more in adults, and 1–2 mL/kg per h or more in children.
2016	Rajeev B.	ISBI Practice Guidelines for Burn Care	**Recommendation:** 1. Adult patients with burns >20% TBSA, and pediatric patients with burns >10% TBSA, should be formally resuscitated with salt-containing fluids. 2. When IV fluid administration is practical, between 2 and 4 mL/kg body weight/burn surface area (% total body surface area, TBSA) should be administered within the first 24 h after injury, with alertness to over-resuscitation. 3. If only oral fluid administration is practical, drinking liquids (typical of the local diet) equivalent to 15% of the body weight every 24 h is recommended for 2 days. **Objective:** For adults, titrate provided fluids to average patients' urine outputs of 0.3–0.5 mL/kg/h; in children titrate to 1 mL/kg/h.
2023	Cartotto R	American burn association clinical practice guidelines on burn shock resuscitation.	**Recommendation:** 1. We recommend that clinicians consider starting acute fluid resuscitation using 2 mL/kg/%TBSA burn to reduce the total volume of resuscitation fluids. 2. We recommend that clinicians consider providing albumin in the first 24 h of resuscitation to improve urinary output and to reduce the total volume of resuscitation fluids. 3. We recommend that initiation of albumin any time in the first 12 h preferably be considered in the context of a research study. 4. We recommend that FFP be used in acute burn shock resuscitation only in the context of a research study.
**Sepsis guidelines**			
2004	R. Phillip Dellinger	Surviving sepsis campaign guidelines for management of severe sepsis and septic shock	**Recommendation:** 1. Fluid resuscitation may consist of natural or artificial colloids or crystalloids. 2. Fluid challenge in patients with suspected hypovolemia may be given at a rate of 500–1,000 mL of crystalloids or 300–500 mL of colloids over 30 min and repeated based on response and tolerance. 3. During the first 6 h of resuscitation of severe sepsis or septic shock, if ScvO_2_ or SVO_2_ of 70% is not achieved with fluid resuscitation to a central venous pressure of 8–12 mm Hg, then transfuse packed red blood cells to achieve a hematocrit of ≥30% and/or administer a dobutamine infusion to achieve this goal. **Objective:** CVP: 8–12 mm Hg; MAP≥65 mm Hg; Urine output>0.5 mL·kg/h; ScvO_2_ or SVO_2_≥70%.
2008	R. Phillip Dellinger	Surviving sepsis campaign: international guidelines for management of severe sepsis and septic shock: 2008	**Recommendation:** 1. We recommend fluid resuscitation with either natural/artificial colloids or crystalloids. 2. We recommend that fluid resuscitation initially target a CVP of>8 mm Hg (12 mmHg in mechanically ventilated patients). 3a. We recommend that a fluid challenge technique be applied where in fluid administration is continued as long as the hemodynamic improvement continues. 3b. We recommend that fluid challenge in patients with suspected hypovolemia be started with ≥1,000 mL of crystalloids or 300–500 mL of colloids over 30 min. 3c. We recommend that the rate of fluid administration be reduced substantially when cardiac filling pressures (CVP or pulmonary artery balloon-occluded pressure) increase without concurrent hemodynamic improvement. 4. We suggest that during the first 6 h of resuscitation of severe sepsis or septic shock, if ScvO_2_ or SVO_2_ of 70 or 65%, respectively, is not achieved with fluid resuscitation to the CVP target, then transfusion of packed red blood cells to achieve a hematocrit of ≥30% and/or administration of a dobutamine infusion be used to achieve this goal. **Objective:** CVP 8–12 mmHg; MAP≥65 mm Hg; Urine output≥0.5 mL/kg/h; ScvO_2_ or SVO_2_ ≥70% or≥65%, respectively.
2012	R. Phillip Dellinger	Surviving sepsis campaign: international guidelines for management of severe sepsis and septic shock, 2012	**Recommendation:** 1. Crystalloids as the initial fluid of choice in the resuscitation of severe sepsis and septic shock. 2. Against the use of hydroxyethyl starches for fluid resuscitation of severe sepsis and septic shock. 3. Albumin in the fluid resuscitation of severe sepsis and septic shock when patients require substantial amounts of crystalloids. 4. Initial fluid challenge in patients with sepsis-induced tissue hypoperfusion with suspicion of hypovolemia to achieve a minimum of 30mL/kg of crystalloids. More rapid administration and greater amounts of fluid may be needed in some patients. 5. Fluid challenge technique be applied wherein fluid administration is continued as long as there is hemodynamic improvement either based on dynamic or static variables. **Objective:** CVP 8–12 mmHg; MAP≥65 mmHg; Urine output≥0.5 mL kg h-1; ScvO_2_ or SVO_2_ ≥70 or≥65%, respectively. 2. We suggest targeting resuscitation to normalize lactate in patients with elevated lactate levels as a marker of tissue hypoperfusion.
2016	Andrew Rhodes	Surviving sepsis campaign: international guidelines for management of sepsis and septic shock: 2016	**Recommendation:** 1. We recommend that a fluid challenge technique be applied where fluid administration is continued as long as hemodynamic factors continue to improve. 2. We recommend crystalloids as the fluid of choice for initial resuscitation and subsequent intravascular volume replacement in patients with sepsis and septic shock. 3. We suggest using either balanced crystalloids or saline for fluid resuscitation of patients with sepsis or septic shock. 4. We suggest using albumin in addition to crystalloids for initial resuscitation and subsequent intravascular volume replacement in patients with sepsis and septic shock when patients require substantial amounts of crystalloids. 5. We recommend against using HESs for intravascular volume replacement in patients with sepsis or septic shock. 6. We suggest using crystalloids over gelatins when resuscitating patients with sepsis or septic shock.
2021	Evans L	Surviving sepsis: campaign: international guidelines for management of sepsis and septic shock 2021	**Recommendation:** 1. For patients with sepsis induced hypoperfusion or septic shock we suggest that at least 30 mL/kg of intravenous (IV) crystalloid fluid should be given within the first 3 h of resuscitation. 2. we suggest using dynamic measures to guide fluid resuscitation, over physical examination or static parameters alone. **Objective:** 1. we suggest guiding resuscitation to decrease serum lactate in patients with elevated lactate level, over not using serum lactate. 2. we suggest using capillary refill time to guide resuscitation as an adjunct to other measures of perfusion. 3. we recommend an initial target MAP of 65 mm Hg over higher MAP targets.

ScvO_2_, central venous oxygen saturation; SVO_2_, mixed venous oxygen saturation; CVP, Central venous pressure; MAP, Mean arterial pressure; HESs, hydroxyethyl starches; TBSA, total burn surface area; FFP, fresh frozen plasma.

We found that the Surviving Sepsis Campaign guidelines for the management of severe sepsis and septic shock have been updated 5 times from 2004 to 2021 (Table 3). The recent updates recommend that at least 30 mL/kg of intravenous (IV) crystalloid fluid be administered within the first 3 h of resuscitation and suggest the use of dynamic measures to guide individualized fluid resuscitation in patients with sepsis-induced hypoperfusion (18).

## Discussion

This bibliometric analysis demonstrated that high-frequency keywords were similarly concentrated in the clusters of “outcomes and complications” as well as “titration of fluid therapy” in publications of fluid resuscitation in patients with either sepsis or burns after 1992. Similarly, mortality ranked the top in the occurrence of the keywords, followed with the keywords about overload associated organ dysfunction or complications, such as AKI, acute respiratory distress syndrome (ARDS), fluid creep, and ACS, etc. in the cluster of “outcomes and complications.” However, our findings suggested that the research priorities were different to the methods of optimizing fluids for sepsis and burns. It was found that the high-frequency keywords and highly cited articles with regard to fluid titration were more tightly linked to targeting hemodynamics improvement in sepsis resuscitation. Meanwhile, the results that “Parkland formula” ranked the 8th of high-frequency keywords, with 4 out of the top 10 frequently cited articles related to “formula” improvement suggested that use of formula to limit resuscitation volume was more emphasized in initial resuscitation for burns. Accordingly, it could be helpful for further researches in titrating fluids for patients with either sepsis or burns to understand the rationales for these diverse research interests.

It has long been recognized that fluid overload leads to worsened organ dysfunction and outcomes in critically ill patients (35). Meanwhile, unnecessary positive fluid balance remained common while resuscitating patients with sepsis (36, 37) or burn shock (9, 38–41). Actually, a great effort has been taken in striking the right fluid balance between adequate and over resuscitation in sepsis for decades, including titration with fluid responsiveness static or dynamic measurements such as central venous pressure (CVP), stroke volume variation (SVV), pulse pressure variation (PPV) and passive leg raising (PLR) (42); or hemodynamic endpoints such as capillary refill time (CRT), lactate clearance, central venous saturation, Pv-a CO_2_ gap and urinary output (43). Similarly, research priority was given to titrate fluids in initial resuscitation for burn shock, such as investigating use of malperfusion markers (such as lactate, base deficit and urine output), or hemodynamic endpoints (CVP, transpulmonary thermodilution-derived variables, or arterial waveform analysis) (44) or algorithm-based and computer supported decisions to optimize fluid resusciatation (45, 46). However, the optimal strategy of fluid titration for these patients remains uncertain. Researches with regard to maximizing benefits while minimizing adverse effects in fluid resuscitation are urgently needed for patients with sepsis or burn shock.

The strength of this study was that a different priority was found between researches on titrating fluids for sepsis and burn shock. Generally, the research priority was tightly related to the distinctive manifestations of a critical illness, for instance, the increased microvascular permeability in burns and the hemodynamic instability in sepsis. However, it could be a limitation that the diverse pathophysiological alternations of resulting in hypovolemia in a specific critical illness such as sepsis or burns have not been well weighed in previous researches of titrating fluids.

Optimization of hemodynamic endpoint has been the focus of sepsis research since the Chicago conference updated the definition of sepsis (47). For instance, early goal directed therapy (EGDT) ranked first in the fluid optimization among the top 20 keywords (Table 1) and the original article was cited most frequently in the retrieved sepsis publications (Table 2) (14). In addition, fluid responsiveness, restrictive fluid management and individualizing resuscitation strategies have been comprehensively studied in the past two decades (43, 48). Although the ideal strategy remains inconclusive, it was emphasized that the fluid administration should be tailored to the patient's individual needs, either “by time” or “by amount” in sepsis resuscitation (49). Meanwhile, these approaches of titrating fluids for sepsis were largely based on the transient effects on increase of cardiac output or tissue perfusion improvement, despite of a concern about organ dysfunction associated with excessive fluids. Pathophysiological alternations in microvasculature such as the intensity of endothelium injury has been under investigated in fluid titration for sepsis (50). Significantly, dynamic assessments on hemodynamics plus intensity of endothelium injury (or microvascular permeability) would be a novelty in further research, and beneficial to the efficiency and safety in clinical practices of fluid resuscitation for sepsis.

Being the fundamental pathophysiological alternation (51), differently, the increased microvascular permeability was seriously concerned in initial fluids resuscitation for burn shock. As showed in Table 1, the keywords of “microvascular exchange” ranked at the second place in burns publications. “Parkland formula” and “formula” ranked the 8th and the 14th of high-frequency keywords, with 4 out of the top 10 frequently cited articles related to “formula” improvement (Tables 1, 3; Supplementary Table S1) suggested that microvascular permeability involved fluid resuscitation strategy was widely investigated (52, 53), although the severity of microvascular permeability was simply estimated by the percentage of TBSA burn. In addition, assessment of hemodynamic endpoint, such as Pulse index Continuous Cardiac Output (PiCCO) that was demonstrated useful for optimizing initial fluid therapy in sepsis, have been attempted to guide titration of fluid requirement for severe burn too (24, 54, 55). However, the findings were controversial. Importantly, it was reported that equivalent to higher fluid resuscitation volumes was administrated in severe burn patients driven by invasive monitoring device than formulas or standard resuscitation, which is often guided by urine volume. Moreover, administration of albumin in initial fluid resuscitation is a hot spot in the field of fluid type domain in burn literature (Table 1), and aims to reduce the total amount of fluid resuscitation and increase urine output in burn patients while reducing edema-related complications as well. Due to insufficient evidence, no recommendation was made on this issue by the currently updated American Burn Association Clinical Practice Guidelines on Burn Shock Resuscitation (32). These findings indicated that the research interests were more likely concentrated in the intensity of endothelium injury (i.e., microvascular permeability) rather than in circulatory function (i.e., hemodynamical instability) in fluid titration for burns. Meanwhile, hemodynamical instability is the major subsequence of hypovolemia, which should be used as the important goal of fluid therapy in severe burns. Based on our results (i.e., the puzzles regarding resuscitation target on hemodynamics), using hemodynamics as a percentage of TBSA based restrictive goal could be a new attempt in further researches on fluid resuscitation in burns.

Naturally, it is complex to decide how much fluids a critically ill patient including burn or sepsis requires individually. First, the causes of hypovolemia were diverse not only between burns and sepsis, but among individuals. The second, fluid response was not identical among the tested patients. Finally, we could hardly estimate the dynamic depletion of fluid in individuals of either burns or sepsis. Therefore, multidimensional integration of characteristics of a critical illness, such as sepsis or burns (comprising of the severity of circulation failure, microvasculature injury, the physiological responsiveness to fluids, etc.) should logically ensure effective and safe fluid resuscitation in either clinical practices or further trials.

This study has at least three limitations. First, the database used in this study is the Web of Science Core Collection database (WOSCC). Failure to include literature related to fluid resuscitation for burns and sepsis not included by WOSCC, such as literature in Chinese, Japanese or German may have affected the results. Second, the frequency of citation was time dependent (i.e., the latest publications would be less likely citated). Therefore, there was a limitation to evaluate the importance of a publication with the citations. Moreover, among 468 burn publications, 12 keywords with very low-frequency ( ≤ 30) were in the top 20 high-frequency keywords, indicating that the topic concentration of burn fluid resuscitation was not high. Therefore, low-frequency keywords, although ranking in top 20, should be carefully interpreted.

## Conclusions

It was demonstrated that the research priorities in titrating fluids were different, while concerning of “outcomes and complications” similarly in fluid resuscitation for patients with burns and sepsis after 1992. Researches were more likely concentrated on targeting hemodynamics in sepsis, but on improving formula to calculate the degree of capillary leakage in burns resuscitation. This difference could be attributable to their specifically important pathophysiological alterations, i.e., hemodynamic instability in sepsis vs. the increased microvascular permeability in burns. However, these pathophysiological alternations have been simultaneously considered in few previous studies, although both of them were important (but with different weight) causes for hypovolemia in either sepsis or burns.

## Supplementary material

Supplementary Figure S1 Cumulative proportions of publications on fluid resuscitation for burns and sepsis from 1992 to 2022. The X-axis represented the year from 1992 to 2022, the Y-axis represented the cumulative proportions of publications on fluid resuscitation for burn (dark bar) and sepsis (gray bar). (Cumulative proportions of publications on fluid resuscitation for either burn or sepsis was calculated as the cumulative publications on fluid resuscitation/all publications on either burns or sepsis from 1992 till 2022).

Supplementary Figure S2 Analysis of the co-citation network of cited references. The node's size indicated the frequency of citations; the larger the node, the higher the number of references cited. In Figure **(A, B**) showed the mapping of the co-citation in publications on fluid resuscitation for sepsis and burn, respectively.
