# Supplementary figures and images for: Lessons from the similarities and differences in fluid resuscitation between burns and sepsis: a bibliometric analysis
Source: Front Med (Lausanne). 2025 Mar 4;12:1561619. doi: 10.3389/fmed.2025.1561619 (PMC11914137; doi:10.3389/fmed.2025.1561619)

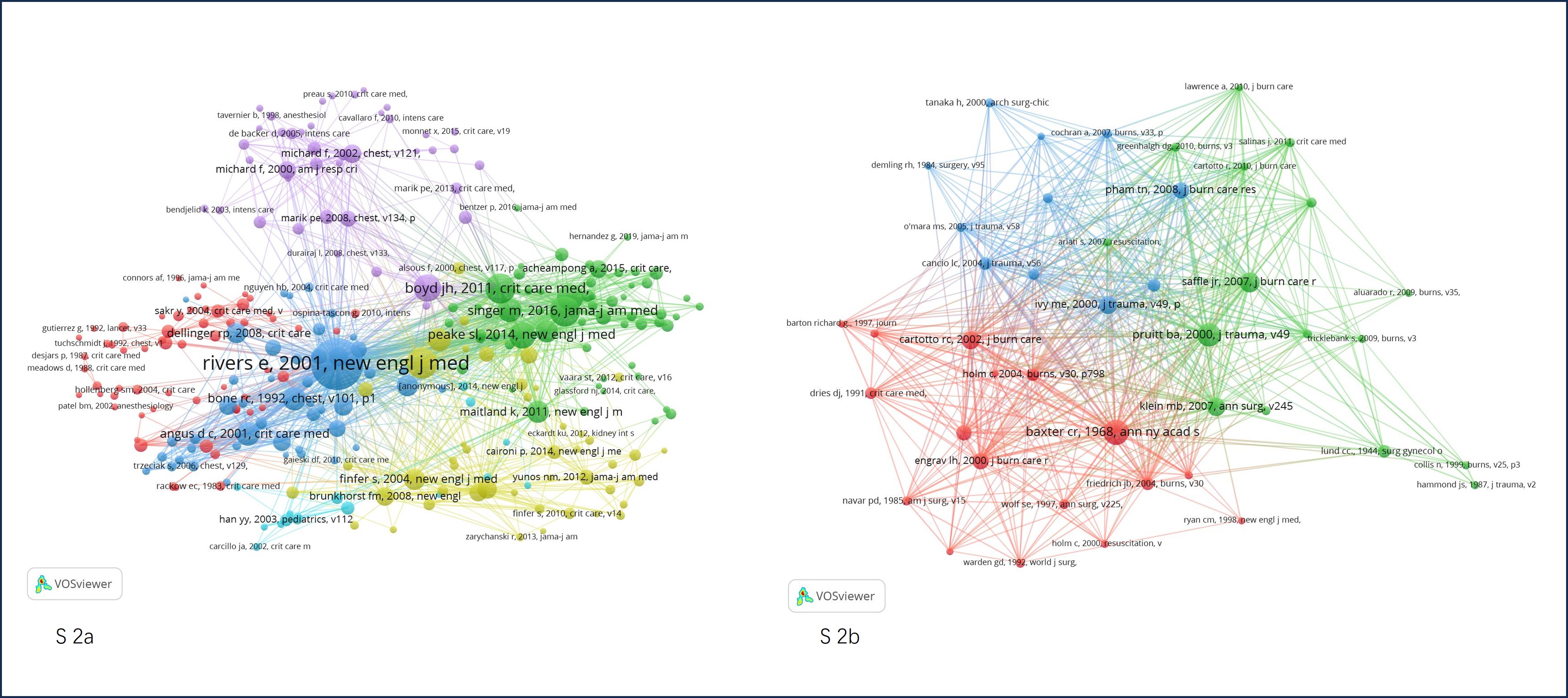

Supplement: Supplementary Figure S2 — Analysis of the co-citation network of cited references. The node's size indicated the frequency of citations; the larger the node, the higher the number of references cited. In Figure (A, B) showed the mapping of the co-citation in publications on fluid resuscitation for sepsis and burn, respectively. [file Image_2.JPEG]
