# Supplementary material for: Lessons from the similarities and differences in fluid resuscitation between burns and sepsis: a bibliometric analysis
Source: Front Med (Lausanne). 2025 Mar 4;12:1561619. doi: 10.3389/fmed.2025.1561619 (PMC11914137; doi:10.3389/fmed.2025.1561619)
Supplement: Supplementary file 3 [file Table_1.DOCX]

**Table S1: Keywords with more than 5 co-occurrences in publications on fluid resuscitation for burn and sepsis**

| **Clusters** | **Sepsis** | | | | |  | **Clusters** | **Burn** | | | | |
| --- | --- | --- | --- | --- | --- | --- | --- | --- | --- | --- | --- | --- |
|  | **AAY** | **Rank** | **Keywords** | **Occurrences** | **Total link strength** |  |  | **AAY** | **Rank** | **Keywords** | **Occurrences** | **Total link strength** |
| 1 | 2011.2 | 17 | Vascular dysfunction | 76 | 361 |  | 1 | 2010.1 | 2 | Microvascular exchange | 61 | 178 |
| 1 | 2012.1 | 30 | Acute lung injury | 47 | 236 |  | 1 | 2009.4 | 15 | Blood pressure | 22 | 54 |
| 1 | 2008.5 | 37 | Nitric-oxide | 42 | 220 |  | 1 | 2003.6 | 23 | Rats | 16 | 40 |
| 1 | 2013.6 | 41 | Respiratory-distress-syndrome | 41 | 192 |  | 1 | 2010.5 | 24 | Volume | 16 | 57 |
| 1 | 2012.4 | 47 | Trial | 35 | 147 |  | 1 | 2007.3 | 28 | Edema | 14 | 50 |
| 1 | 2012.1 | 48 | Acute-renal-failure | 33 | 153 |  | 1 | 2004.6 | 29 | Permeability | 14 | 43 |
| 1 | 2014.3 | 49 | Inflammation | 33 | 152 |  | 1 | 2008 | 33 | Vitamin c | 13 | 41 |
| 1 | 2009.4 | 50 | Mechanisms | 33 | 158 |  | 1 | 2002.3 | 34 | Skin | 12 | 40 |
| 1 | 2008.1 | 54 | Endotoxemia | 31 | 171 |  | 1 | 2011 | 35 | Ascorbic acid | 11 | 31 |
| 1 | 2013.5 | 57 | Hydrocortisone | 29 | 122 |  | 1 | 2015.5 | 36 | Inflammation | 11 | 29 |
| 1 | 2002.8 | 63 | Tumor-necrosis-factor | 27 | 107 |  | 1 | 2005.1 | 39 | Multiple organ failure | 10 | 39 |
| 1 | 2013.2 | 64 | Heart-failure | 26 | 112 |  | 1 | 2006.9 | 40 | Nitric-oxide | 10 | 30 |
| 1 | 2009.2 | 68 | Hypertonic saline | 25 | 127 |  | 1 | 2009.2 | 44 | Oral rehydration therapy | 9 | 18 |
| 1 | 2009.2 | 76 | Expression | 23 | 111 |  | 1 | 2006.1 | 49 | Lipid-peroxidation | 8 | 31 |
| 1 | 2007.3 | 87 | Inhibition | 20 | 100 |  | 1 | 2005.8 | 51 | Plasma | 8 | 25 |
| 1 | 2006.6 | 90 | Nitric-oxide synthase | 20 | 105 |  | 1 | 2001.6 | 53 | Sheep | 8 | 29 |
| 1 | 2006.9 | 91 | Rats | 20 | 101 |  | 1 | 2011.6 | 63 | Enteral resuscitation | 6 | 10 |
| 1 | 2018 | 96 | Endothelial glycocalyx | 19 | 109 |  | 1 | 2006.1 | 64 | Hypertonic saline | 6 | 20 |
| 1 | 2008.3 | 99 | Myocardial depression | 19 | 114 |  | 1 | 2005.1 | 72 | Xanthine-oxidase | 6 | 18 |
| 1 | 2014.4 | 100 | Oxidative stress | 19 | 84 |  | 1 | 2020 | 75 | Endothelial glycocalyx | 5 | 17 |
| 1 | 2009.1 | 104 | Activated protein-c | 18 | 87 |  | 1 | 2013.8 | 77 | Hydroxyethyl starch | 5 | 17 |
| 1 | 2006.1 | 105 | Lipopolysaccharide | 18 | 80 |  | 1 | 2007.4 | 78 | Infusion | 5 | 18 |
| 1 | 2011.1 | 106 | Permeability | 18 | 105 |  | 1 | 2010.4 | 79 | Oxidative stress | 5 | 18 |
| 1 | 2011.2 | 108 | Vasopressin | 18 | 115 |  | 1 | 1996 | 82 | Protein flux | 5 | 15 |
| 1 | 2011.1 | 110 | Arginine-vasopressin | 17 | 96 |  | 1 | 2009.8 | 83 | Responses | 5 | 19 |
| 1 | 2017.5 | 121 | Prognosis | 16 | 84 |  | 1 | 2001.4 | 85 | Tissue | 5 | 13 |
| 1 | 2012 | 123 | Corticosteroids | 15 | 60 |  | 2 | 2006.4 | 9 | Cardiac-output | 26 | 86 |
| 1 | 2010.6 | 124 | Double-blind | 15 | 78 |  | 2 | 2005.1 | 18 | Oxygen delivery | 20 | 73 |
| 1 | 2010.4 | 127 | Endothelium | 14 | 67 |  | 2 | 2011.7 | 20 | Serum lactate | 17 | 69 |
| 1 | 2008.4 | 131 | Porcine model | 14 | 86 |  | 2 | 2012.2 | 21 | Shock resuscitation | 17 | 72 |
| 1 | 2014 | 133 | Transfusion | 14 | 63 |  | 2 | 2011.8 | 22 | Survival | 17 | 60 |
| 1 | 2011.1 | 137 | Circulatory shock | 13 | 78 |  | 2 | 2008.6 | 26 | Crystalloid resuscitation | 15 | 72 |
| 1 | 2006.8 | 139 | Cytokines | 13 | 54 |  | 2 | 2010.2 | 27 | Base deficit | 14 | 67 |
| 1 | 2010.3 | 140 | Heart | 13 | 72 |  | 2 | 2008.6 | 41 | Randomized trial | 10 | 30 |
| 1 | 2012.6 | 144 | Myocardial dysfunction | 13 | 66 |  | 2 | 2010.7 | 42 | Smoke-inhalation | 10 | 41 |
| 1 | 2005.5 | 145 | Nitric oxide | 13 | 77 |  | 2 | 2011 | 48 | Intrathoracic blood-volume | 8 | 35 |
| 1 | 2000.8 | 146 | Rat | 13 | 71 |  | 2 | 2011.8 | 54 | Thermodilution | 8 | 40 |
| 1 | 2006.5 | 150 | Depression | 12 | 69 |  | 2 | 2007.1 | 55 | Extravascular lung water | 7 | 39 |
| 1 | 2009.9 | 154 | Intensive insulin therapy | 12 | 68 |  | 2 | 2007.7 | 56 | Hemodynamics | 7 | 26 |
| 1 | 2009.2 | 160 | Terlipressin | 12 | 72 |  | 2 | 2009.5 | 61 | Pulmonary-artery catheter | 7 | 26 |
| 1 | 2019.2 | 165 | Glycocalyx | 11 | 45 |  | 2 | 2002.5 | 62 | Critically ill patients | 6 | 20 |
| 1 | 2011.8 | 168 | Mice | 11 | 37 |  | 2 | 2013.1 | 66 | Morbidity | 6 | 30 |
| 1 | 2011.6 | 170 | Plasma | 11 | 49 |  | 2 | 2013.5 | 67 | Organ dysfunction | 6 | 27 |
| 1 | 2014.9 | 172 | Strategies | 11 | 58 |  | 2 | 2011.6 | 69 | Protein | 6 | 16 |
| 1 | 2006.6 | 181 | Renal function | 10 | 55 |  | 2 | 2007.2 | 74 | End-points | 5 | 20 |
| 1 | 2010.8 | 187 | Acute renal failure | 9 | 36 |  | 3 | 2013.3 | 1 | Mortality | 63 | 206 |
| 1 | 2007.5 | 189 | Contractility | 9 | 59 |  | 3 | 2011.4 | 3 | Inhalation injury | 54 | 168 |
| 1 | 2013.2 | 197 | Mitochondrial dysfunction | 9 | 50 |  | 3 | 2011.6 | 4 | Sepsis | 46 | 164 |
| 1 | 2006.3 | 200 | Pathogenesis | 9 | 37 |  | 3 | 2012.3 | 5 | ACS | 41 | 132 |
| 1 | 2007.6 | 203 | Vascular permeability | 9 | 46 |  | 3 | 2015.8 | 10 | Acute kidney injury | 26 | 82 |
| 1 | 2007.2 | 205 | Adrenal insufficiency | 8 | 31 |  | 3 | 2015.9 | 13 | Outcomes | 24 | 81 |
| 1 | 2012.6 | 209 | Cardiac dysfunction | 8 | 47 |  | 3 | 2007.3 | 30 | Dysfunction | 13 | 40 |
| 1 | 2015.8 | 215 | Esmolol | 8 | 43 |  | 3 | 2012.1 | 37 | Epidemiology | 10 | 29 |
| 1 | 2014.5 | 219 | Pathophysiology | 8 | 33 |  | 3 | 2010.1 | 47 | Failure | 8 | 24 |
| 1 | 2010.7 | 220 | Physiology | 8 | 41 |  | 3 | 2014 | 50 | Multicenter | 8 | 27 |
| 1 | 2001.8 | 222 | Responses | 8 | 35 |  | 3 | 2015 | 57 | Infection | 7 | 18 |
| 1 | 2003.2 | 225 | Swine | 8 | 32 |  | 3 | 2014.2 | 58 | Intensive-care-unit | 7 | 19 |
| 1 | 2015.5 | 227 | Accumulation | 7 | 37 |  | 3 | 2017.1 | 60 | Prognosis | 7 | 27 |
| 1 | 2013.5 | 231 | Controlled-trial | 7 | 44 |  | 3 | 2001.8 | 65 | Ischemia | 6 | 19 |
| 1 | 2009.8 | 232 | Disseminated intravascular coagulation | 7 | 26 |  | 3 | 2010.3 | 68 | Pediatric burns | 6 | 22 |
| 1 | 2004.5 | 234 | Factor-alpha | 7 | 41 |  | 3 | 2010.2 | 76 | Fluid balance | 5 | 20 |
| 1 | 2013.4 | 237 | Levosimendan | 7 | 45 |  | 3 | 2013.2 | 81 | Prevention | 5 | 11 |
| 1 | 2012.8 | 239 | Murine model | 7 | 39 |  | 3 | 2011.6 | 84 | Severity | 5 | 18 |
| 1 | 2003 | 242 | Pulmonary | 7 | 37 |  | 3 | 2013.8 | 86 | Transfusion | 5 | 12 |
| 1 | 2003.2 | 243 | Sheep | 7 | 38 |  | 4 | 2014.6 | 6 | Management | 40 | 118 |
| 1 | 2000 | 244 | Small-volume resuscitation | 7 | 23 |  | 4 | 2014 | 7 | Critical care | 33 | 98 |
| 1 | 2013.4 | 245 | Systemic inflammation | 7 | 34 |  | 4 | 2015.5 | 11 | Fluid creep | 25 | 82 |
| 1 | 2011.3 | 248 | Advanced Vasodilatory shock | 6 | 39 |  | 4 | 2017 | 16 | Accuracy | 21 | 47 |
| 1 | 2011.8 | 250 | Atrial-natriuretic-Peptide | 6 | 34 |  | 4 | 2017.4 | 17 | Burn size | 21 | 42 |
| 1 | 2010.8 | 251 | Cortisol | 6 | 22 |  | 4 | 2014.3 | 31 | Emergency | 13 | 32 |
| 1 | 2010.1 | 253 | Cytokine | 6 | 27 |  | 4 | 2016.3 | 32 | TBSA | 13 | 26 |
| 1 | 2009.3 | 254 | Diastolic dysfunction | 6 | 37 |  | 4 | 2019.5 | 43 | Major burn | 9 | 27 |
| 1 | 2015.1 | 255 | Early goal directed therapy | 6 | 26 |  | 4 | 2012.7 | 45 | Respiratory-distress-syndrome | 9 | 35 |
| 1 | 2014.6 | 259 | End-expiratory pressure | 6 | 30 |  | 4 | 2012.6 | 46 | Acute lung injury | 8 | 24 |
| 1 | 2003.3 | 261 | Gene-expression | 6 | 22 |  | 4 | 2015.8 | 59 | Metabolism | 7 | 5 |
| 1 | 2008.5 | 268 | Interleukin-6 | 6 | 14 |  | 4 | 2016 | 80 | Prehospital | 5 | 16 |
| 1 | 2007 | 270 | Intravital microscopy | 6 | 33 |  | 5 | 2014.5 | 8 | Parkland formula | 32 | 97 |
| 1 | 2003.5 | 273 | Muscle | 6 | 21 |  | 5 | 2012.8 | 12 | Colloid resuscitation | 25 | 102 |
| 1 | 2009.1 | 275 | Necrosis-factor-alpha | 6 | 37 |  | 5 | 2011 | 14 | Formula | 23 | 70 |
| 1 | 2009.5 | 276 | Neutrophils | 6 | 34 |  | 5 | 2012.8 | 19 | Albumin | 20 | 58 |
| 1 | 2007.6 | 281 | Pulmonary-edema | 6 | 41 |  | 5 | 2011.1 | 25 | Burn shock | 15 | 48 |
| 1 | 2015 | 283 | Receptor | 6 | 39 |  | 5 | 2014.3 | 38 | Guidelines | 10 | 31 |
| 1 | 2012.8 | 284 | Renal-failure | 6 | 32 |  | 5 | 2011.3 | 52 | Requirements | 8 | 31 |
| 1 | 2017.1 | 287 | Stress | 6 | 39 |  | 5 | 2010.5 | 70 | Saline | 6 | 22 |
| 1 | 2020 | 290 | Thiamine | 6 | 23 |  | 5 | 2016.2 | 71 | Urine output | 6 | 22 |
| 1 | 2014.1 | 293 | Vasodilatory shock | 6 | 28 |  | 5 | 2005 | 73 | Complications | 5 | 15 |
| 1 | 2009 | 298 | Adhesion | 5 | 32 |  |  |  |  |  |  |  |
| 1 | 2001 | 305 | Cardiovascular dysfunction | 5 | 26 |  |  |  |  |  |  |  |
| 1 | 2011.8 | 312 | Down-regulation | 5 | 13 |  |  |  |  |  |  |  |
| 1 | 2013 | 313 | Early fluid resuscitation | 5 | 22 |  |  |  |  |  |  |  |
| 1 | 2008.8 | 316 | Hypertonic saline resuscitation | 5 | 27 |  |  |  |  |  |  |  |
| 1 | 2009.6 | 317 | Low-dose vasopressin | 5 | 32 |  |  |  |  |  |  |  |
| 1 | 2016 | 319 | Mean arterial-pressure | 5 | 28 |  |  |  |  |  |  |  |
| 1 | 2001.4 | 321 | Monoclonal-antibody | 5 | 15 |  |  |  |  |  |  |  |
| 1 | 2004.6 | 322 | Oxide synthase inhibition | 5 | 24 |  |  |  |  |  |  |  |
| 1 | 2009.2 | 325 | Peroxynitrite | 5 | 29 |  |  |  |  |  |  |  |
| 1 | 2012 | 327 | Platelets | 5 | 27 |  |  |  |  |  |  |  |
| 1 | 2013.8 | 329 | Pneumonia | 5 | 31 |  |  |  |  |  |  |  |
| 1 | 2014.8 | 333 | Septic shock patients | 5 | 31 |  |  |  |  |  |  |  |
| 1 | 2020.2 | 339 | Vitamin c | 5 | 20 |  |  |  |  |  |  |  |
| 2 | 2016 | 4 | Fluid responsiveness | 225 | 1155 |  |  |  |  |  |  |  |
| 2 | 2009.5 | 13 | Cardiac output | 107 | 540 |  |  |  |  |  |  |  |
| 2 | 2012.6 | 15 | Blood pressure | 77 | 389 |  |  |  |  |  |  |  |
| 2 | 2011.9 | 20 | Volume | 69 | 357 |  |  |  |  |  |  |  |
| 2 | 2012.1 | 25 | Mechanically ventilated patients | 57 | 302 |  |  |  |  |  |  |  |
| 2 | 2015.5 | 26 | Echocardiography | 56 | 296 |  |  |  |  |  |  |  |
| 2 | 2015.6 | 27 | Central venous pressure | 55 | 301 |  |  |  |  |  |  |  |
| 2 | 2013.6 | 29 | Blood-pressure | 48 | 272 |  |  |  |  |  |  |  |
| 2 | 2014.2 | 32 | Stroke volume variation | 47 | 276 |  |  |  |  |  |  |  |
| 2 | 2012.4 | 39 | Arterial pressure | 41 | 243 |  |  |  |  |  |  |  |
| 2 | 2017.7 | 42 | Ultrasonography | 38 | 209 |  |  |  |  |  |  |  |
| 2 | 2017 | 44 | Inferior vena-cava | 36 | 187 |  |  |  |  |  |  |  |
| 2 | 2012 | 46 | Respiratory changes | 35 | 202 |  |  |  |  |  |  |  |
| 2 | 2012.2 | 55 | Extravascular lung water | 31 | 204 |  |  |  |  |  |  |  |
| 2 | 2012.2 | 58 | Transpulmonary thermodilution | 29 | 182 |  |  |  |  |  |  |  |
| 2 | 2014.7 | 59 | Doppler ultrasound | 28 | 152 |  |  |  |  |  |  |  |
| 2 | 2017.1 | 62 | Pulse pressure variation | 27 | 162 |  |  |  |  |  |  |  |
| 2 | 2005.5 | 65 | Performance | 26 | 152 |  |  |  |  |  |  |  |
| 2 | 2015.6 | 72 | Fluid challenge | 24 | 132 |  |  |  |  |  |  |  |
| 2 | 2014.8 | 74 | Vena-cava diameter | 24 | 152 |  |  |  |  |  |  |  |
| 2 | 2017.1 | 78 | Hemodynamic Monitoring | 22 | 111 |  |  |  |  |  |  |  |
| 2 | 2017.9 | 79 | Index | 22 | 94 |  |  |  |  |  |  |  |
| 2 | 2017.3 | 80 | Passive leg raising | 22 | 131 |  |  |  |  |  |  |  |
| 2 | 2010.3 | 81 | Pulmonary-artery catheter | 22 | 141 |  |  |  |  |  |  |  |
| 2 | 2013.2 | 93 | Volume expansion | 20 | 116 |  |  |  |  |  |  |  |
| 2 | 2016.6 | 101 | Parameters | 19 | 112 |  |  |  |  |  |  |  |
| 2 | 2010.1 | 102 | Ventilated patients | 19 | 106 |  |  |  |  |  |  |  |
| 2 | 2015.9 | 114 | Prediction | 17 | 87 |  |  |  |  |  |  |  |
| 2 | 2010.8 | 115 | Preload | 17 | 101 |  |  |  |  |  |  |  |
| 2 | 2017.2 | 116 | Respiratory variation | 17 | 96 |  |  |  |  |  |  |  |
| 2 | 2012 | 118 | ARDS | 16 | 96 |  |  |  |  |  |  |  |
| 2 | 2012 | 128 | Hypovolemia | 14 | 96 |  |  |  |  |  |  |  |
| 2 | 2007.4 | 129 | Intrathoracic blood-volume | 14 | 86 |  |  |  |  |  |  |  |
| 2 | 2014 | 134 | Blood-volume | 13 | 76 |  |  |  |  |  |  |  |
| 2 | 2012.3 | 135 | Cardiac index | 13 | 69 |  |  |  |  |  |  |  |
| 2 | 2016 | 136 | Challenge | 13 | 61 |  |  |  |  |  |  |  |
| 2 | 2008.6 | 138 | Clinical-trials | 13 | 71 |  |  |  |  |  |  |  |
| 2 | 2015 | 142 | Marker | 13 | 60 |  |  |  |  |  |  |  |
| 2 | 2011.9 | 143 | Monitoring | 13 | 81 |  |  |  |  |  |  |  |
| 2 | 2015.5 | 152 | Fluid management | 12 | 58 |  |  |  |  |  |  |  |
| 2 | 2015.6 | 159 | Risk-factors | 12 | 50 |  |  |  |  |  |  |  |
| 2 | 2013.8 | 162 | Venous return | 12 | 50 |  |  |  |  |  |  |  |
| 2 | 2010.6 | 167 | Indicator | 11 | 64 |  |  |  |  |  |  |  |
| 2 | 2008 | 171 | Pulmonary edema | 11 | 62 |  |  |  |  |  |  |  |
| 2 | 2011 | 173 | Transesophageal echocardiography | 11 | 72 |  |  |  |  |  |  |  |
| 2 | 2013.3 | 174 | Acute respiratory distress syndrome | 10 | 69 |  |  |  |  |  |  |  |
| 2 | 2016.6 | 180 | Predictor | 10 | 52 |  |  |  |  |  |  |  |
| 2 | 2016.4 | 186 | Volume status | 10 | 48 |  |  |  |  |  |  |  |
| 2 | 2009.1 | 191 | Fluid loading | 9 | 47 |  |  |  |  |  |  |  |
| 2 | 2016.4 | 192 | Haemodynamics | 9 | 41 |  |  |  |  |  |  |  |
| 2 | 2018 | 193 | Heart failure | 9 | 41 |  |  |  |  |  |  |  |
| 2 | 2008.6 | 195 | Hypovolemic shock | 9 | 42 |  |  |  |  |  |  |  |
| 2 | 2018.7 | 207 | Bioreactance | 8 | 42 |  |  |  |  |  |  |  |
| 2 | 2005.7 | 210 | Circulation | 8 | 37 |  |  |  |  |  |  |  |
| 2 | 2004.6 | 213 | Dogs | 8 | 40 |  |  |  |  |  |  |  |
| 2 | 2018.5 | 216 | Fluid bolus | 8 | 37 |  |  |  |  |  |  |  |
| 2 | 2013.8 | 217 | Hospital stay | 8 | 44 |  |  |  |  |  |  |  |
| 2 | 2013.1 | 221 | Randomized controlled-trial | 8 | 34 |  |  |  |  |  |  |  |
| 2 | 2015.5 | 223 | Right atrial pressure | 8 | 44 |  |  |  |  |  |  |  |
| 2 | 2010.1 | 230 | Cardiac preload | 7 | 44 |  |  |  |  |  |  |  |
| 2 | 2009.1 | 233 | End-diastolic volume | 7 | 47 |  |  |  |  |  |  |  |
| 2 | 2014.4 | 246 | Tidal volume | 7 | 41 |  |  |  |  |  |  |  |
| 2 | 2001.6 | 257 | Ejection fraction | 6 | 35 |  |  |  |  |  |  |  |
| 2 | 2019.1 | 258 | End-expiratory occlusion | 6 | 47 |  |  |  |  |  |  |  |
| 2 | 2014.5 | 262 | Guide | 6 | 29 |  |  |  |  |  |  |  |
| 2 | 2013.3 | 263 | Heart-lung interactions | 6 | 32 |  |  |  |  |  |  |  |
| 2 | 2010.1 | 266 | Hypertension | 6 | 30 |  |  |  |  |  |  |  |
| 2 | 2006.8 | 267 | Indocyanine green | 6 | 32 |  |  |  |  |  |  |  |
| 2 | 2018.8 | 269 | Intraabdominal hypertension | 6 | 27 |  |  |  |  |  |  |  |
| 2 | 2019 | 278 | Point-of-care ultrasound | 6 | 29 |  |  |  |  |  |  |  |
| 2 | 2012.5 | 280 | Predicts fluid responsiveness | 6 | 36 |  |  |  |  |  |  |  |
| 2 | 2014.1 | 286 | Shock index | 6 | 33 |  |  |  |  |  |  |  |
| 2 | 2009.1 | 288 | Systolic pressure variation | 6 | 28 |  |  |  |  |  |  |  |
| 2 | 2017.6 | 291 | Transthoracic echocardiography | 6 | 32 |  |  |  |  |  |  |  |
| 2 | 2016.3 | 295 | Water | 6 | 25 |  |  |  |  |  |  |  |
| 2 | 2011 | 309 | Consensus conference | 5 | 31 |  |  |  |  |  |  |  |
| 2 | 2015.4 | 315 | Heart rate | 5 | 34 |  |  |  |  |  |  |  |
| 2 | 2016.4 | 318 | Lung ultrasound | 5 | 29 |  |  |  |  |  |  |  |
| 2 | 2016.2 | 328 | Pleth variability index | 5 | 30 |  |  |  |  |  |  |  |
| 2 | 2012.4 | 331 | Pulse contour analysis | 5 | 29 |  |  |  |  |  |  |  |
| 2 | 2015.8 | 335 | Spontaneously breathing patients | 5 | 35 |  |  |  |  |  |  |  |
| 2 | 2017.4 | 340 | Volume overload | 5 | 21 |  |  |  |  |  |  |  |
| 3 | 2016.3 | 1 | Mortality | 426 | 1772 |  |  |  |  |  |  |  |
| 3 | 2016.5 | 2 | Management | 331 | 1507 |  |  |  |  |  |  |  |
| 3 | 2016 | 3 | Goal directed Resuscitation | 239 | 1112 |  |  |  |  |  |  |  |
| 3 | 2017.3 | 8 | Outcome | 124 | 513 |  |  |  |  |  |  |  |
| 3 | 2017.6 | 10 | Fluid balance※ | 120 | 534 |  |  |  |  |  |  |  |
| 3 | 2012 | 11 | Organ dysfunction | 118 | 542 |  |  |  |  |  |  |  |
| 3 | 2014.3 | 14 | Survival | 87 | 418 |  |  |  |  |  |  |  |
| 3 | 2014.6 | 19 | Multicenter | 70 | 327 |  |  |  |  |  |  |  |
| 3 | 2018.4 | 22 | Fluid overload | 66 | 325 |  |  |  |  |  |  |  |
| 3 | 2016.3 | 33 | Definitions | 45 | 186 |  |  |  |  |  |  |  |
| 3 | 2015.2 | 35 | Hemodynamic support | 43 | 154 |  |  |  |  |  |  |  |
| 3 | 2011.8 | 36 | Hypotension | 43 | 248 |  |  |  |  |  |  |  |
| 3 | 2014.3 | 38 | Surviving sepsis campaign | 42 | 182 |  |  |  |  |  |  |  |
| 3 | 2015.3 | 51 | Protocol | 33 | 140 |  |  |  |  |  |  |  |
| 3 | 2013.1 | 53 | Venous oxygen-saturation | 32 | 188 |  |  |  |  |  |  |  |
| 3 | 2013.4 | 56 | Implementation | 31 | 125 |  |  |  |  |  |  |  |
| 3 | 2015.7 | 60 | Fluid administration | 28 | 150 |  |  |  |  |  |  |  |
| 3 | 2015.5 | 66 | Vasoactive drugs | 26 | 155 |  |  |  |  |  |  |  |
| 3 | 2014.4 | 71 | Early goal-directed therapy | 24 | 122 |  |  |  |  |  |  |  |
| 3 | 2017 | 77 | Bundles | 22 | 94 |  |  |  |  |  |  |  |
| 3 | 2020.6 | 82 | Campaign international guidelines | 21 | 109 |  |  |  |  |  |  |  |
| 3 | 2016.4 | 88 | Lactate clearance | 20 | 102 |  |  |  |  |  |  |  |
| 3 | 2016.2 | 94 | Clinical-practice parameters | 19 | 58 |  |  |  |  |  |  |  |
| 3 | 2016.2 | 109 | antimicrobial therapy | 17 | 71 |  |  |  |  |  |  |  |
| 3 | 2018.4 | 119 | Biomarker | 16 | 74 |  |  |  |  |  |  |  |
| 3 | 2018.8 | 122 | Prognostic-factor | 16 | 96 |  |  |  |  |  |  |  |
| 3 | 2017.3 | 125 | International guidelines | 15 | 72 |  |  |  |  |  |  |  |
| 3 | 2014.6 | 147 | Serum lactate | 13 | 65 |  |  |  |  |  |  |  |
| 3 | 2014 | 149 | Clearance | 12 | 59 |  |  |  |  |  |  |  |
| 3 | 2015.7 | 151 | Early lactate clearance | 12 | 64 |  |  |  |  |  |  |  |
| 3 | 2016 | 155 | Intravenous fluids | 12 | 73 |  |  |  |  |  |  |  |
| 3 | 2017.3 | 158 | Positive fluid balance | 12 | 67 |  |  |  |  |  |  |  |
| 3 | 2012.2 | 183 | System | 10 | 59 |  |  |  |  |  |  |  |
| 3 | 2012.3 | 199 | Output | 9 | 43 |  |  |  |  |  |  |  |
| 3 | 2012.7 | 211 | Clinical-practice | 8 | 32 |  |  |  |  |  |  |  |
| 3 | 2013.5 | 224 | Sofa score | 8 | 43 |  |  |  |  |  |  |  |
| 3 | 2015 | 260 | Failure assessment score | 6 | 36 |  |  |  |  |  |  |  |
| 3 | 2015.6 | 264 | Heart-rate | 6 | 26 |  |  |  |  |  |  |  |
| 3 | 2006.8 | 277 | Noradrenaline | 6 | 43 |  |  |  |  |  |  |  |
| 3 | 2016.1 | 279 | Predictors | 6 | 21 |  |  |  |  |  |  |  |
| 3 | 2017.5 | 282 | Quality improvement | 6 | 17 |  |  |  |  |  |  |  |
| 3 | 2011 | 285 | Risk-factor | 6 | 20 |  |  |  |  |  |  |  |
| 3 | 2018.3 | 289 | Tachycardia | 6 | 32 |  |  |  |  |  |  |  |
| 3 | 2015.8 | 292 | Treatment | 6 | 31 |  |  |  |  |  |  |  |
| 3 | 2010 | 303 | Cardiac-arrest | 5 | 24 |  |  |  |  |  |  |  |
| 3 | 2010.8 | 304 | Cardiopulmonary variables | 5 | 21 |  |  |  |  |  |  |  |
| 3 | 2018 | 308 | Clinical-outcomes | 5 | 18 |  |  |  |  |  |  |  |
| 3 | 2017 | 314 | Emergency medical services | 5 | 12 |  |  |  |  |  |  |  |
| 3 | 2017.2 | 330 | Procalcitonin | 5 | 27 |  |  |  |  |  |  |  |
| 3 | 2017.6 | 332 | Reduced mortality | 5 | 32 |  |  |  |  |  |  |  |
| 3 | 2017.4 | 338 | Systemic inflammatory response | 5 | 29 |  |  |  |  |  |  |  |
| 3 | 2018 | 341 | Water-electrolyte balance | 5 | 13 |  |  |  |  |  |  |  |
| 4 | 2009.9 | 6 | Microcirculatory Blood-flow | 140 | 713 |  |  |  |  |  |  |  |
| 4 | 2010.9 | 9 | Norepinephrine therapy | 122 | 685 |  |  |  |  |  |  |  |
| 4 | 2009.5 | 12 | Hemodynamic | 110 | 629 |  |  |  |  |  |  |  |
| 4 | 2013.3 | 23 | Lactate | 62 | 304 |  |  |  |  |  |  |  |
| 4 | 2003.6 | 24 | Oxygen delivery | 61 | 329 |  |  |  |  |  |  |  |
| 4 | 2013.2 | 28 | Perfusion | 55 | 291 |  |  |  |  |  |  |  |
| 4 | 2002.5 | 31 | Oxygen consumption | 47 | 271 |  |  |  |  |  |  |  |
| 4 | 2004.2 | 34 | Dobutamine | 43 | 279 |  |  |  |  |  |  |  |
| 4 | 2007.5 | 40 | Dopamine | 41 | 234 |  |  |  |  |  |  |  |
| 4 | 2009.6 | 69 | Tissue oxygenation | 25 | 137 |  |  |  |  |  |  |  |
| 4 | 2011.7 | 73 | Vasopressors | 24 | 143 |  |  |  |  |  |  |  |
| 4 | 2009.9 | 75 | Epinephrine | 23 | 146 |  |  |  |  |  |  |  |
| 4 | 2011.1 | 83 | Flow | 21 | 98 |  |  |  |  |  |  |  |
| 4 | 2002 | 84 | Catecholamines | 20 | 140 |  |  |  |  |  |  |  |
| 4 | 2018.6 | 85 | Consensus | 20 | 85 |  |  |  |  |  |  |  |
| 4 | 2007.2 | 89 | Multiple organ failure | 20 | 122 |  |  |  |  |  |  |  |
| 4 | 1995.5 | 92 | Respiratory-distress syndrome | 20 | 91 |  |  |  |  |  |  |  |
| 4 | 2012.3 | 107 | Severity | 18 | 87 |  |  |  |  |  |  |  |
| 4 | 2008.1 | 111 | Inflammatory response syndrome | 17 | 79 |  |  |  |  |  |  |  |
| 4 | 2002 | 112 | Intramucosal ph | 17 | 111 |  |  |  |  |  |  |  |
| 4 | 2010.4 | 117 | Animal model | 16 | 83 |  |  |  |  |  |  |  |
| 4 | 2002.7 | 132 | Tonometry | 14 | 102 |  |  |  |  |  |  |  |
| 4 | 2009.3 | 141 | Hypoperfusion | 13 | 71 |  |  |  |  |  |  |  |
| 4 | 1996.9 | 148 | Therapeutic goals | 13 | 69 |  |  |  |  |  |  |  |
| 4 | 2002.9 | 153 | Gastric intramucosal PH | 12 | 79 |  |  |  |  |  |  |  |
| 4 | 2013 | 157 | Oxygenation | 12 | 70 |  |  |  |  |  |  |  |
| 4 | 2007.7 | 161 | Variables | 12 | 62 |  |  |  |  |  |  |  |
| 4 | 2009.5 | 163 | Acidosis | 11 | 48 |  |  |  |  |  |  |  |
| 4 | 2014.3 | 166 | Hypoxia | 11 | 40 |  |  |  |  |  |  |  |
| 4 | 2018 | 169 | Peripheral perfusion | 11 | 53 |  |  |  |  |  |  |  |
| 4 | 2003.1 | 177 | Low-dose dopamine | 10 | 69 |  |  |  |  |  |  |  |
| 4 | 2005.1 | 178 | Pigs | 10 | 56 |  |  |  |  |  |  |  |
| 4 | 2017.7 | 182 | Saturation | 10 | 51 |  |  |  |  |  |  |  |
| 4 | 2000.2 | 184 | Transport | 10 | 58 |  |  |  |  |  |  |  |
| 4 | 2014.8 | 185 | Vasoactive agents | 10 | 58 |  |  |  |  |  |  |  |
| 4 | 2014.1 | 196 | Microcirculatory alterations | 9 | 54 |  |  |  |  |  |  |  |
| 4 | 2001.8 | 201 | Regional blood flow | 9 | 51 |  |  |  |  |  |  |  |
| 4 | 2010.5 | 204 | Acute circulatory failure | 8 | 47 |  |  |  |  |  |  |  |
| 4 | 2005.2 | 218 | Oxygen | 8 | 42 |  |  |  |  |  |  |  |
| 4 | 2013.7 | 226 | Tissue hypoxia | 8 | 40 |  |  |  |  |  |  |  |
| 4 | 2018.5 | 228 | Capillary refill time | 7 | 35 |  |  |  |  |  |  |  |
| 4 | 1998.2 | 235 | Hyperdynamic sepsis | 7 | 37 |  |  |  |  |  |  |  |
| 4 | 2001.2 | 241 | Oxygen extraction | 7 | 51 |  |  |  |  |  |  |  |
| 4 | 2006.1 | 249 | Apache-II | 6 | 22 |  |  |  |  |  |  |  |
| 4 | 2014 | 256 | Edema | 6 | 35 |  |  |  |  |  |  |  |
| 4 | 2015.1 | 265 | Hyperlactatemia | 6 | 32 |  |  |  |  |  |  |  |
| 4 | 2003.8 | 271 | Maximizing oxygen delivery | 6 | 34 |  |  |  |  |  |  |  |
| 4 | 2012.8 | 274 | Near-infrared spectroscopy | 6 | 29 |  |  |  |  |  |  |  |
| 4 | 2012.5 | 294 | Volume resuscitation | 6 | 32 |  |  |  |  |  |  |  |
| 4 | 2001.2 | 299 | Adrenaline | 5 | 28 |  |  |  |  |  |  |  |
| 4 | 2009.4 | 300 | Base deficit | 5 | 27 |  |  |  |  |  |  |  |
| 4 | 2013 | 320 | Microvascular perfusion | 5 | 23 |  |  |  |  |  |  |  |
| 4 | 2001.2 | 323 | Oxygen transport | 5 | 24 |  |  |  |  |  |  |  |
| 4 | 2005.2 | 326 | PH | 5 | 23 |  |  |  |  |  |  |  |
| 4 | 2006.6 | 334 | Splanchnic perfusion | 5 | 38 |  |  |  |  |  |  |  |
| 4 | 2010.6 | 337 | Sublingual capnometry | 5 | 25 |  |  |  |  |  |  |  |
| 5 | 2017.2 | 5 | Acute kidney injury | 148 | 764 |  |  |  |  |  |  |  |
| 5 | 2013 | 7 | Hydroxyethyl starch | 128 | 651 |  |  |  |  |  |  |  |
| 5 | 2015.8 | 16 | Sodium chloride | 77 | 370 |  |  |  |  |  |  |  |
| 5 | 2012.2 | 18 | Albumin | 70 | 361 |  |  |  |  |  |  |  |
| 5 | 2011.7 | 21 | Colloids | 67 | 390 |  |  |  |  |  |  |  |
| 5 | 2016.3 | 43 | Fluid | 36 | 174 |  |  |  |  |  |  |  |
| 5 | 2013.2 | 45 | Crystalloids | 35 | 231 |  |  |  |  |  |  |  |
| 5 | 2010.3 | 52 | Infusion | 32 | 168 |  |  |  |  |  |  |  |
| 5 | 2011.1 | 61 | Renal failure | 28 | 126 |  |  |  |  |  |  |  |
| 5 | 2020.4 | 67 | Balanced crystalloids | 25 | 132 |  |  |  |  |  |  |  |
| 5 | 2015.9 | 70 | 0.9-percent saline | 24 | 150 |  |  |  |  |  |  |  |
| 5 | 2011.1 | 86 | Gelatin | 20 | 98 |  |  |  |  |  |  |  |
| 5 | 2015.2 | 95 | Crystalloid | 19 | 128 |  |  |  |  |  |  |  |
| 5 | 2017 | 97 | Hyperchloremia | 19 | 120 |  |  |  |  |  |  |  |
| 5 | 2011.8 | 98 | Inflammatory response | 19 | 91 |  |  |  |  |  |  |  |
| 5 | 2009.5 | 103 | Volume therapy | 19 | 89 |  |  |  |  |  |  |  |
| 5 | 2004.4 | 113 | Lactic-acidosis | 17 | 93 |  |  |  |  |  |  |  |
| 5 | 2014.3 | 120 | Chloride | 16 | 115 |  |  |  |  |  |  |  |
| 5 | 2016.9 | 126 | Renal blood-flow | 15 | 92 |  |  |  |  |  |  |  |
| 5 | 2012.7 | 130 | Metabolic acidosis | 14 | 84 |  |  |  |  |  |  |  |
| 5 | 2017.3 | 156 | Kidney injury | 12 | 64 |  |  |  |  |  |  |  |
| 5 | 2015.3 | 164 | Fluid-management | 11 | 54 |  |  |  |  |  |  |  |
| 5 | 2016.6 | 175 | Hyperchloremic acidosis | 10 | 64 |  |  |  |  |  |  |  |
| 5 | 2017 | 176 | Lactated ringers solution | 10 | 68 |  |  |  |  |  |  |  |
| 5 | 2010.7 | 179 | Plasma-volume | 10 | 44 |  |  |  |  |  |  |  |
| 5 | 2012.2 | 188 | Capillary leakage | 9 | 50 |  |  |  |  |  |  |  |
| 5 | 2008.3 | 190 | Dextran | 9 | 45 |  |  |  |  |  |  |  |
| 5 | 2002.6 | 194 | Hetastarch | 9 | 51 |  |  |  |  |  |  |  |
| 5 | 2015 | 198 | Normal saline | 9 | 47 |  |  |  |  |  |  |  |
| 5 | 2011.6 | 202 | Serum-albumin | 9 | 42 |  |  |  |  |  |  |  |
| 5 | 2018.5 | 206 | Balanced solutions | 8 | 41 |  |  |  |  |  |  |  |
| 5 | 2017.5 | 208 | Bolus | 8 | 23 |  |  |  |  |  |  |  |
| 5 | 2014.2 | 212 | Complications | 8 | 49 |  |  |  |  |  |  |  |
| 5 | 2012 | 214 | Efficacy | 8 | 39 |  |  |  |  |  |  |  |
| 5 | 2007.4 | 229 | Capillary-permeability | 7 | 30 |  |  |  |  |  |  |  |
| 5 | 2013.1 | 236 | Hyperoncotic colloids | 7 | 34 |  |  |  |  |  |  |  |
| 5 | 2008.8 | 238 | Modified fluid gelatin | 7 | 38 |  |  |  |  |  |  |  |
| 5 | 2014.2 | 240 | Organ function | 7 | 35 |  |  |  |  |  |  |  |
| 5 | 2012.8 | 247 | Acid-base | 6 | 40 |  |  |  |  |  |  |  |
| 5 | 2016.1 | 252 | Creatinine | 6 | 30 |  |  |  |  |  |  |  |
| 5 | 2009.5 | 272 | Microvascular permeability | 6 | 26 |  |  |  |  |  |  |  |
| 5 | 2013.4 | 296 | 6-percent hydroxyethyl starch | 5 | 22 |  |  |  |  |  |  |  |
| 5 | 2009.2 | 297 | Acid-base balance | 5 | 28 |  |  |  |  |  |  |  |
| 5 | 2005 | 301 | Bicarbonate | 5 | 32 |  |  |  |  |  |  |  |
| 5 | 2007.4 | 302 | Capillary leak syndrome | 5 | 34 |  |  |  |  |  |  |  |
| 5 | 2016 | 306 | Circulating inflammatory molecules | 5 | 37 |  |  |  |  |  |  |  |
| 5 | 2012.8 | 307 | Clinical trial | 5 | 27 |  |  |  |  |  |  |  |
| 5 | 2015.8 | 310 | Crystalloid solutions | 5 | 21 |  |  |  |  |  |  |  |
| 5 | 2019.4 | 311 | Death | 5 | 12 |  |  |  |  |  |  |  |
| 5 | 2001 | 324 | Pentastarch | 5 | 34 |  |  |  |  |  |  |  |
| 5 | 2012 | 336 | Starch | 5 | 19 |  |  |  |  |  |  |  |

Occurrences were calculated on co-occurrence frequency of the keywords. Total link strength: Links between nodes can be cooperative relationships, co-occurrence relationships, reference relationships, etc., and each link has a weight or strength value. Total link strength is the sum of the strength of all the links connecting two nodes. AAY: Average appearing years (the average publication year of the articles in which the keyword occurs (to the nearest 1 decimal place)). AKI: Acute kidney injury; ARDS: Acute respiratory distress syndrome. ACS: abdominal compartment syndrome. TBSA: Total body surface area. Fluid balance^※^: Is the result of combining balance with fluid balance.
