# Supplementary material for: Lessons from the similarities and differences in fluid resuscitation between burns and sepsis: a bibliometric analysis
Source: Front Med (Lausanne). 2025 Mar 4;12:1561619. doi: 10.3389/fmed.2025.1561619 (PMC11914137; doi:10.3389/fmed.2025.1561619)
Supplement: Supplementary file 4 [file Table_2.DOCX]

**Table S2: The top 10 latest vs earliest keywords in publications on Fluid Resuscitation for Sepsis and Burn.**

| **Rank** | **Sepsis** | | | | | **Rank** | **Burn** | | | | |
| --- | --- | --- | --- | --- | --- | --- | --- | --- | --- | --- | --- |
|  | **AAY** | **Keywords** | **Occurrences** | | **Total link strength** |  | **AAY** | **Keywords** | **Occurrences** | | **Total link strength** |
| **Latest** | | | | | | **Latest** | | | | | |
| 1 | 2016.3 | Mortality | | 426 | 1772 | 1 | 2014.6 | Management | | 40 | 118 |
| 2 | 2016.5 | Management | | 331 | 1507 | 2 | 2014.0 | Critical care | | 33 | 98 |
| 3 | 2016.0 | Goal directed resuscitation | | 239 | 1112 | 3 | 2014.5 | Parkland formula | | 32 | 97 |
| 4 | 2016.0 | Fluid responsiveness | | 225 | 1155 | 4 | 2015.8 | Acute kidney injury | | 26 | 82 |
| 5 | 2017.2 | Acute kidney injury | | 148 | 764 | 5 | 2015.5 | Fluid creep | | 25 | 82 |
| 6 | 2017.3 | Outcome | | 124 | 513 | 6 | 2015.9 | Outcomes | | 24 | 81 |
| 7 | 2014.3 | Survival | | 87 | 418 | 7 | 2017.4 | Burn size | | 21 | 42 |
| 8 | 2015.8 | Sodium chloride | | 77 | 370 | 8 | 2017.0 | Accuracy | | 21 | 47 |
| 9 | 2014.6 | Multicenter | | 70 | 327 | 9 | 2016.3 | TBSA | | 13 | 26 |
| 10 | 2017.1 | Balance | | 68 | 332 | 10 | 2014.3 | Emergency | | 13 | 32 |
| **Earliest** | | | | | | **Earliest** | | | | | |
| 1 | 2003.6 | Oxygen delivery | | 61 | 329 | 1 | 2006.4 | Cardiac-output | | 26 | 86 |
| 2 | 2002.5 | Oxygen consumption | | 47 | 271 | 2 | 2005.1 | Oxygen delivery | | 20 | 73 |
| 3 | 2004.2 | Dobutamine | | 43 | 279 | 3 | 2003.6 | Rats | | 16 | 40 |
| 4 | 2002.8 | Tumor-necrosis-factor | | 27 | 107 | 4 | 2004.6 | Permeability | | 14 | 43 |
| 5 | 2005.5 | Performance | | 26 | 152 | 5 | 2002.3 | Skin | | 12 | 40 |
| 6 | 1995.5 | ARDS | | 20 | 91 | 6 | 2005.1 | Multiple organ failure | | 10 | 39 |
| 7 | 2002.0 | Catecholamines | | 20 | 140 | 7 | 2006.9 | Nitric-oxide | | 10 | 30 |
| 8 | 2006.6 | Nitric-oxide synthase | | 20 | 105 | 8 | 2001.6 | Sheep | | 8 | 29 |
| 9 | 2006.9 | Rats | | 20 | 101 | 9 | 2005.8 | Plasma | | 8 | 25 |
| 10 | 2006.1 | Lipopolysaccharide | | 18 | 80 | 10 | 2006.1 | Lipid-peroxidation | | 8 | 31 |

Latest：After 2014 (The yellow bubble shown in Fig. 3c and Fig. 3d); Earliest: Before 2006 (The blue bubble shown in Fig. 3c and Fig. 3d); ARDS: Acute respiratory distress syndrome; TBSA: Total burn surface area.
